# MyoSpectrumNet: a unified deep learning framework for pathologic myopia spectrum assessment using heterogeneous multi-source fundus datasets

**DOI:** 10.3389/fmed.2026.1881289

**Published:** 2026-10-27

**Authors:** Bo Zhang, Feng Lin, Weidong Li, Biyun Lin

**Affiliations:** 1 Ophthalmology Department, The First Hospital of Putian, Putian, Fujian, China; 2 Mathematics Group, Putian No.3 High School, Putian, Fujian, China

**Keywords:** calibration, deep learning, fundus photography, heterogeneous multi-source learning, lesion-aware interpretation, myopic maculopathy, pathologic myopia, screening

## Abstract

**Introduction:**

Pathologic myopia (PM) is a major cause of irreversible visual impairment, yet existing artificial intelligence studies have largely focused on isolated tasks and single-source datasets. Public fundus datasets relevant to PM provide complementary but only partially aligned supervision, creating a need for a unified framework that can support screening, severity assessment, and lesion interpretation across heterogeneous sources.

**Methods:**

We developed MyoSpectrumNet using HPMI, PALM, and MMAC. The source-conditioned multi-task checkpoint was evaluated on held-out partitions from known sources. Additional task-specific SE and lesion comparators were trained on the official MMAC partitions, with hyperparameters selected only within the training records and single-view inference used for the primary matched-split comparisons. Four-view TTA was retained as a within-MyoSpectrumNet sensitivity analysis.

**Results:**

Under single-view inference, the task-specific baselines and MyoSpectrumNet yielded HPMI PM AUCs of 0.987 and 0.985, PALM PM AUCs of 0.996 and 0.993, and MM-grading QWK values of 0.907 and 0.907, respectively. The ConvNeXt-T SE baseline achieved an MAE of 1.006 D (95% CI 0.873–1.147) and Pearson r of 0.801, versus a MyoSpectrumNet single-view MAE of 1.259 D. The DeepLabV3-ResNet50 lesion baseline achieved an overall Dice of 0.397 (95% CI 0.324–0.468), versus 0.472 for the MyoSpectrumNet interpretation checkpoint under single-view inference. These auxiliary cross-model comparisons remain descriptive because the original MyoSpectrumNet auxiliary outputs were retained only as aggregate summaries.

**Conclusion:**

MyoSpectrumNet integrates PM screening, MM grading, auxiliary SE estimation, and lesion-aware visual evidence within one heterogeneous multi-source framework. Task-specific comparisons illustrate the trade-offs of joint learning: it did not improve every output, while the integrated framework retained multiple clinically complementary outputs in one model. The complete multi-output framework has not been externally validated on an unseen dataset, and no universal task-specific superiority claim is made.

## 1 Introduction

Pathologic myopia (PM) has become an increasingly important cause of irreversible visual impairment worldwide, in parallel with the global rise in myopia and high myopia (1, 2). Importantly, PM is not synonymous with high myopia: current definitions emphasize structural damage in the posterior segment rather than refractive error alone (3–6). The META-PM classification provided a widely adopted framework by defining five categories of myopic maculopathy (MM) and three “plus” lesions, thereby improving the consistency of clinical grading and research reporting (3). Subsequent consensus and review work further highlighted posterior staphyloma as a hallmark lesion and underscored the need for earlier identification of eyes at risk of progression to vision-threatening disease (5–8).

Against this clinical background, artificial intelligence (AI) based on retinal imaging has shown considerable promise for PM-related screening and grading (9–11). The field has progressed from early handcrafted or conventional machine-learning approaches (12, 13) to deep learning systems for PM identification, myopic maculopathy categorization, lesion segmentation, and large-scale screening from fundus photographs (14–19). More recent studies have explored self-supervised learning, ensemble strategies, and explicit morphologic pattern modeling to improve myopic maculopathy recognition or interpretability (20–22). However, despite encouraging accuracy, the current evidence base remains dominated by retrospective and largely single-source studies, with most models optimized for only one task at a time and with limited attention to clinically integrated outputs, cross-dataset transportability, calibration, and lesion-level explanation (9–11, 20–22).

The recent emergence of public datasets has made more rigorous benchmarking possible, but has also exposed a key challenge: clinically relevant supervision is distributed across heterogeneous sources. The PALM dataset (PAthoLogic Myopia Challenge) provides color fundus photographs for PM recognition together with anatomical and lesion annotations (23, 24). The MMAC benchmark extends the problem to MM classification, plus-lesion segmentation, and spherical equivalent (SE) prediction (25). The HPMI dataset further supplies a large public resource for high/pathologic myopia identification (26). At the same time, retinal photographs are known to encode information related not only to disease status but also to refractive characteristics, supporting the feasibility of auxiliary SE estimation from fundus images (27, 28). Taken together, these resources are complementary rather than redundant, but they differ substantially in acquisition settings, source populations, label granularity, and task definitions, making naive data pooling or isolated single-task modeling suboptimal.

In this study, we developed MyoSpectrumNet, a unified deep learning framework for pathologic myopia spectrum assessment using HPMI, PALM, and MMAC (23–26). Rather than addressing PM screening, MM grading, SE estimation, and lesion interpretation as separate problems, we formulated them as complementary tasks within a heterogeneous multi-source learning setting. The framework combines a shared encoder with source-conditioned modulation, a lesion-aware branch, and lesion-guided fusion to exploit complementary annotations while respecting cross-source heterogeneity. We hypothesized that this design would preserve screening-oriented PM discrimination while providing integrated severity, refractive, and lesion-aware outputs. The objective was balanced integration and efficient reuse of complementary supervision, not a prespecified expectation that joint training would significantly improve every individual task.

## 2 Methods

### 2.1 Study design and clinical tasks

This study was designed as a heterogeneous multi-source fundus-learning analysis for pathologic myopia spectrum assessment using three public datasets, namely HPMI, PALM, and MMAC (23–26). Rather than treating pathologic myopia evaluation as a single binary classification task on one curated dataset, we formulated it as a clinically oriented multi-task problem spanning screening, severity stratification, auxiliary refractive-status estimation, and lesion-aware interpretation. The overall rationale was that currently available public datasets for pathologic myopia provide complementary but only partially aligned supervision across these tasks, and that a unified framework is therefore needed to leverage these sources in a clinically meaningful manner. The study overview is shown in Figure 1.

**Figure 1:**
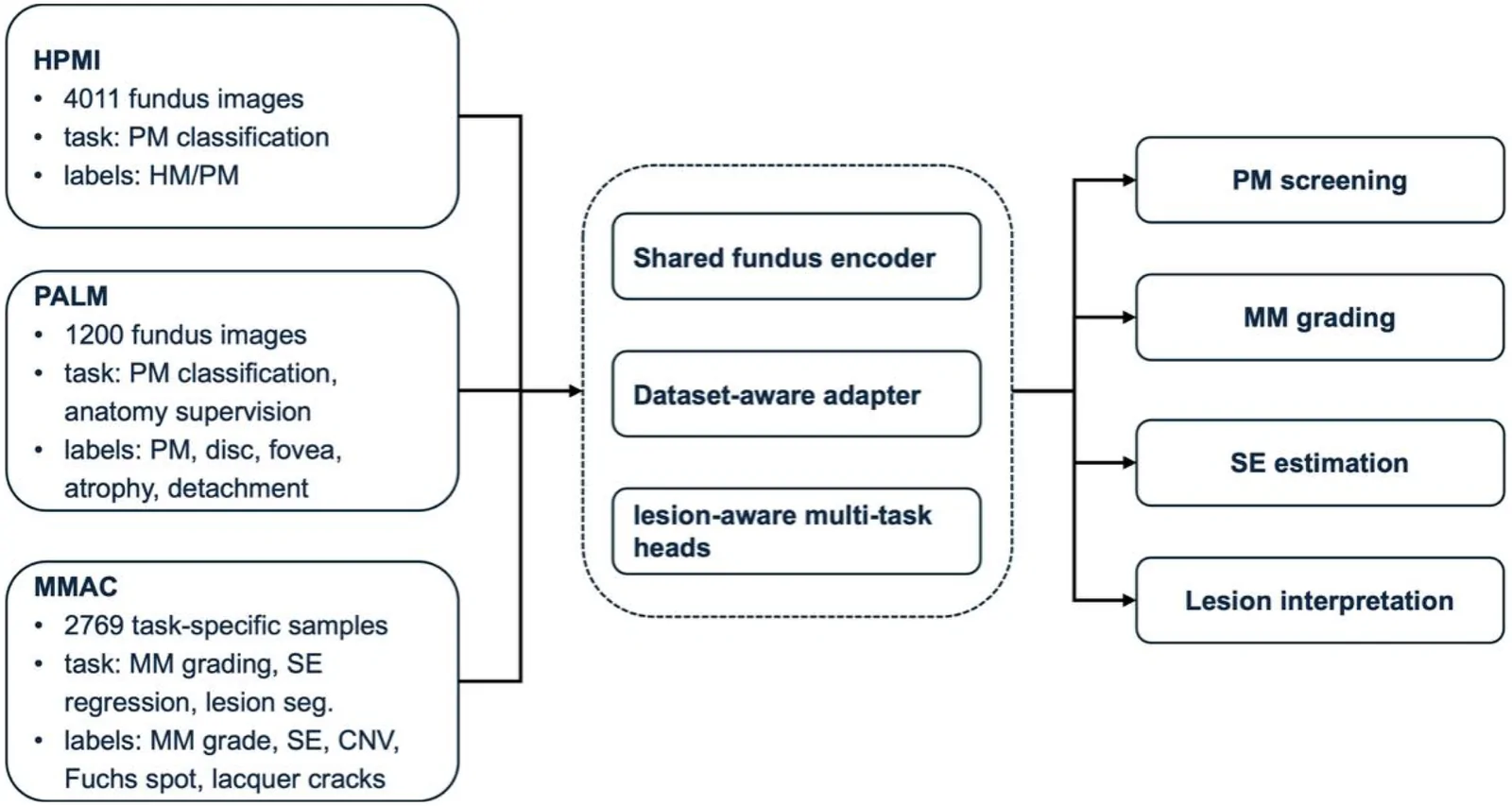
Study overview. Three public fundus datasets with complementary but only partially aligned label spaces were integrated into a unified study design for pathologic myopia spectrum assessment. HPMI provided large-scale PM screening labels, PALM provided PM labels together with anatomy- and lesion-related supervision, and MMAC provided myopic maculopathy grading, spherical equivalent targets, and lesion annotations. These heterogeneous sources were jointly used in MyoSpectrumNet to support PM screening, MM grading, auxiliary SE estimation, and lesion-aware interpretation.

Four tasks were considered in the final study. The primary task was PM screening from color fundus photographs. The secondary tasks were myopic maculopathy grading based on the established META-PM framework (3), auxiliary spherical equivalent estimation from fundus images (25, 27, 28), and lesion-aware interpretation through dense lesion probability maps using available lesion annotations (23, 25). These outputs were intended to reflect the layered clinical workflow of screening, referral support, and disease stratification, rather than to replace ophthalmologist judgment. Binary screening was the main diagnostic output, MM grading and SE estimation enriched severity characterization, and lesion-aware outputs supplied spatial evidence.

Joint prediction was motivated by three considerations: the clinical outputs are complementary within the same referral pathway; the three public datasets contain partially aligned labels that can be used through masked task losses; and a shared encoder can provide all outputs with less computational duplication than four independent deployment models. Accordingly, the principal criterion was whether a single framework could retain screening and grading performance while adding complementary outputs. The protocol-matched single-view point estimates were treated descriptively, and the study does not claim universal benefit over independently optimized task-specific systems.

### 2.2 Datasets, label harmonization, and preprocessing

Three public fundus datasets were included in the final study: HPMI (26), PALM (23, 24), and MMAC (25). HPMI contained 4,011 fundus photographs with a train/validation/test split of 2,406/802/803 and served primarily as a source for PM screening. PALM contained 1,200 images with an official split of 400/400/400 and provided PM labels together with partial anatomy- and lesion-related annotations, including the optic disc, fovea, peripapillary atrophy, and retinal detachment (23, 24). MMAC comprised 2,769 task-specific records and served as the main source for MM grading, auxiliary SE estimation, and lesion-aware supervision, including annotations for choroidal neovascularization (CNV), Fuchs spot, and lacquer cracks (25). Specifically, the MMAC task-specific partitions were 1,143/248 records for MM grading train/validation, 992/205 records for SE regression train/validation, and 149/32 records for lesion segmentation train/validation. A summary of dataset characteristics is provided in Table 1, detailed split and annotation coverage is provided in Supplementary Table S1, and representative examples are shown in Figure 2.

**Table 1:** Characteristics of the datasets included in this study. Counts for HPMI and PALM are image counts; the MMAC value is the sum of task-specific records across grading, SE, and lesion partitions and must not be interpreted as unique images. Detailed task-level counts are provided in Supplementary Table S1.

Dataset	Count basis	Split protocol	Primary tasks	Available annotations	Image size
HPMI	4,011 images	Train/val/test = 2,406/802/803	PM classification	HM/PM binary labels	512 × 512
PALM	1,200 images	Official split = 400/400/400	PM classification and anatomy supervision	PM labels, disc, fovea, atrophy, detachment	2,037 × 1,978 (average)
MMAC	2,769 task-specific records	Task-specific train/validation: MM 1,143/248; SE 992/205; lesion 149/32	MM grading, SE regression, lesion interpretation	MM grade, SE, CNV, Fuchs spot, lacquer cracks	800 × 800

**Figure 2:**
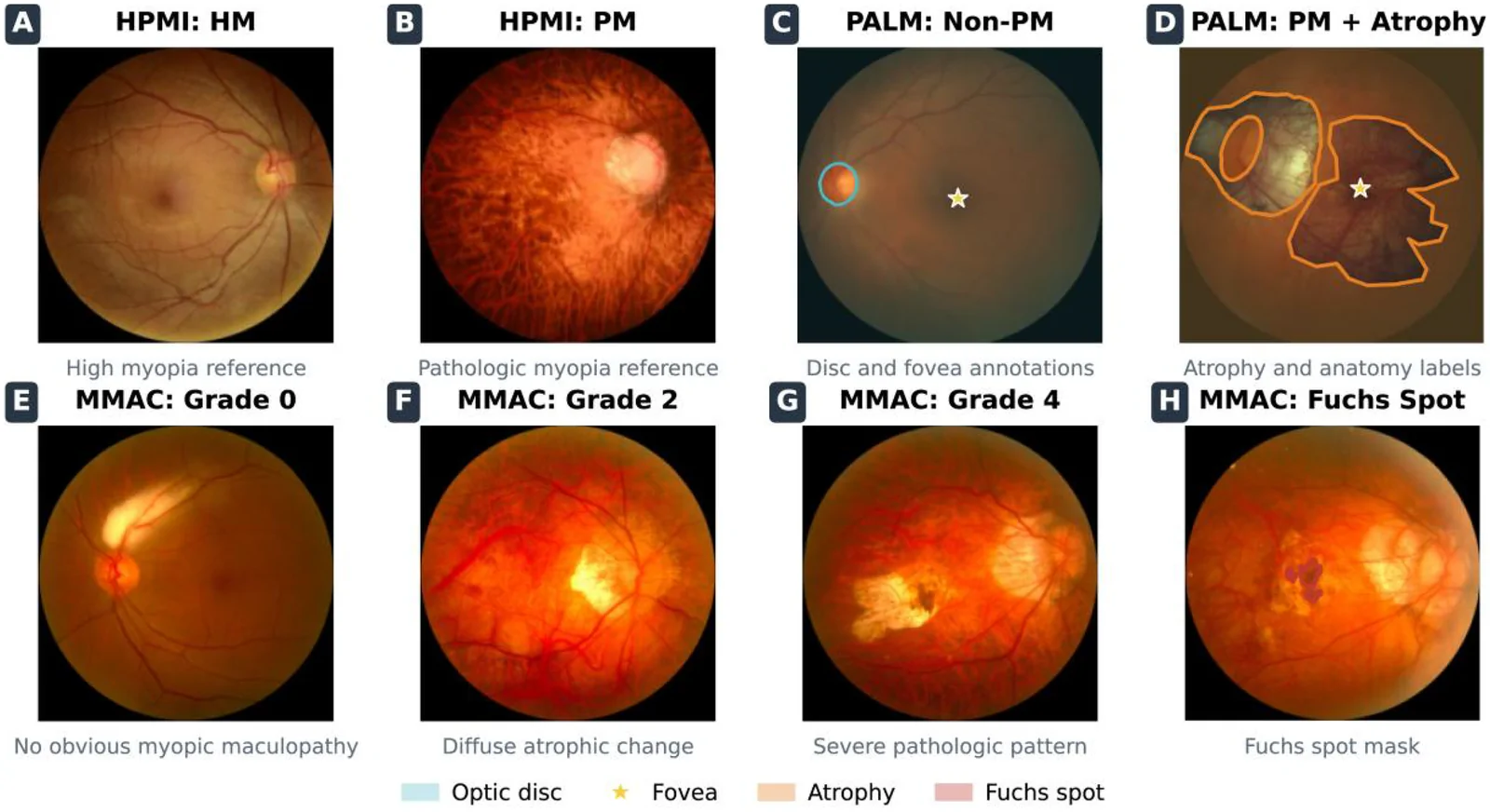
Representative data and label examples. **(A)** A high-myopia (HM) reference image from HPMI. **(B)** A pathologic-myopia (PM) reference image from HPMI. **(C)** A non-PM image from PALM with optic disc and foveal annotations. **(D)** A PM image from PALM with atrophy and anatomical annotations. **(E)** An MMAC myopic maculopathy grade-0 example showing no obvious myopic maculopathy. **(F)** An MMAC grade-2 example showing diffuse atrophic changes. **(G)** An MMAC grade-4 example showing severe pathologic changes. **(H)** An MMAC example with a Fuchs spot segmentation mask. These examples illustrate the diversity of fundus appearances and label types across HPMI, PALM, and MMAC, highlighting the heterogeneous supervision used in the unified learning framework.

Because these datasets differed substantially in source population, annotation protocol, and label granularity, model development was treated as a partially labeled heterogeneous learning problem rather than forcing full label alignment. For PM screening, HPMI distinguished high myopia (HM) from pathologic myopia (PM), whereas PALM distinguished PM from non-PM. Because the negative classes were not equivalent, the PM head learned source-conditioned decision boundaries rather than one source-invariant binary boundary. Each image contributed only to task losses supported by its annotations: HPMI contributed mainly to PM screening; PALM contributed PM screening plus anatomy- and lesion-related cues; and MMAC contributed MM grading, SE estimation, and lesion-aware supervision. All corresponding train/validation/test boundaries were respected. For MMAC, validation records for a task were never used to train that task, and masked losses prevented unannotated outputs from contributing to the objective. All retained models used 384 × 384 inputs. Images were intensity-normalized after loading. Random horizontal flipping, mild rotation, scaling, and color perturbation were used only for training; validation and test images underwent resizing and normalization only, except when explicitly marked as TTA at final inference.

Two evaluation settings were analyzed. First, the principal source-conditioned multi-task checkpoint was trained jointly on the training partitions of HPMI, PALM, and MMAC and evaluated on the corresponding held-out partitions using known dataset identities. This was not a leave-one-dataset-out experiment and does not establish multi-output generalization to an unseen source. Second, Figure 3 and Table 2 report separate PM-only source-to-target controls: a source-specific PM classifier was trained and selected on HPMI or PALM, locked, and evaluated on the other dataset without target labels or fine-tuning. The source-conditioned multi-task adapter was not used in these PM-only controls. Thus, Figure 3 assesses PM-screening transportability under source shift, not unseen-source generalization of the four-output model. Consequently, external generalizability of the complete framework, particularly its MM-grading, SE-estimation, and lesion-aware outputs, remains unestablished.

**Figure 3:**
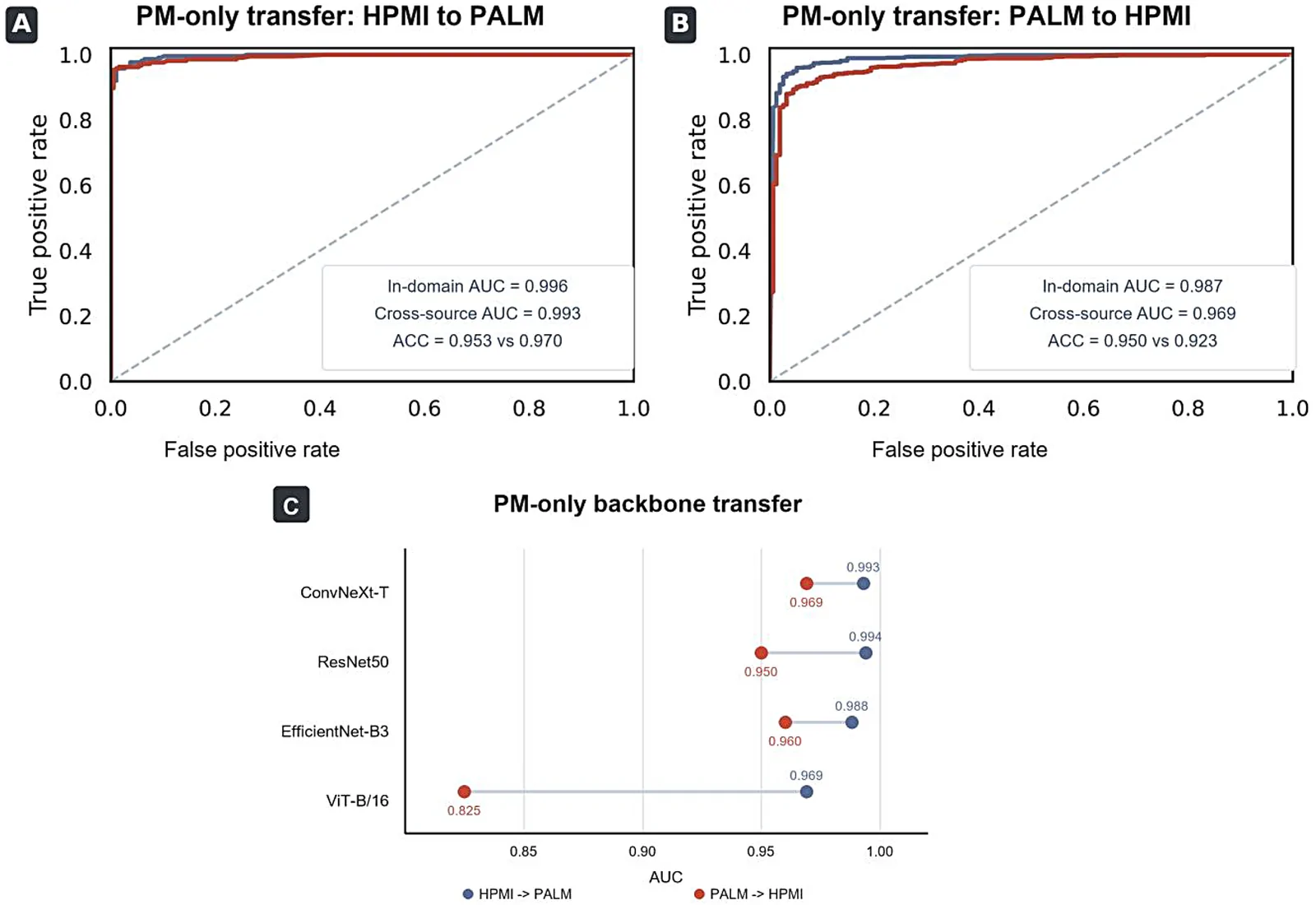
PM-only source-to-target transfer analysis. **(A)** HPMI-to-PALM transfer. **(B)** PALM-to-HPMI transfer. **(C)** AUC comparison across backbones; blue denotes HPMI-to-PALM and red denotes PALM-to-HPMI. These are separately trained source-specific PM classifiers evaluated without target labels or fine-tuning; the source-conditioned multi-task adapter was not used. The analysis does not test unseen-source generalization of the full four-output framework.

**Table 2:** PM-only source-to-target transfer and backbone comparison. Each model was trained and selected on the named source and evaluated on the target without target labels or fine-tuning. The source-conditioned multi-task adapter was not used, and the table does not test the full framework on an unseen source.

Setting	Backbone	AUC	Accuracy	F1 score	Sensitivity	Specificity
HPMI -> PALM (PM-only)	ResNet50	0.994	0.948	0.952	0.981	0.909
HPMI -> PALM (PM-only)	ConvNeXt-T	0.993	0.97	0.971	0.953	0.989
HPMI -> PALM (PM-only)	EfficientNet-B3	0.988	0.948	0.951	0.948	0.947
HPMI -> PALM (PM-only)	ViT-B/16	0.969	0.825	0.858	0.995	0.631
PALM -> HPMI (PM-only)	ConvNeXt-T	0.969	0.923	0.952	0.949	0.813
PALM -> HPMI (PM-only)	EfficientNet-B3	0.96	0.803	0.862	0.764	0.968
PALM -> HPMI (PM-only)	ResNet50	0.95	0.73	0.8	0.668	0.987
PALM -> HPMI (PM-only)	ViT-B/16	0.825	0.813	0.882	0.863	0.606

Because all included datasets were de-identified public resources, patient-level identifiers were not available to directly audit cross-dataset duplication. The risk of substantial overlap was considered low because HPMI, PALM, and MMAC were released from different sources and with different task designs, but a small number of overlapping or near-duplicate fundus photographs across public datasets cannot be completely ruled out. We therefore interpret cross-dataset results as public-dataset transportability tests rather than as fully independent prospective external validation, and this point is explicitly acknowledged as a limitation.

### 2.3 Architecture of MyoSpectrumNet

MyoSpectrumNet was designed as a unified fundus-learning framework with four main components: a shared fundus encoder, a dataset-aware adapter, task-specific prediction heads, and a lesion-aware branch. An overview of the architecture is shown in Figure 4. Given an input image x, a shared encoder E(⋅) first extracted a global visual representation, as defined in Equation 1:

h=E(x) (1)

where h denotes the latent feature embedding. In the retained model, the shared encoder was based on ConvNeXt-T, which provided the most balanced performance across in-domain and cross-dataset analyses.

**Figure 4:**
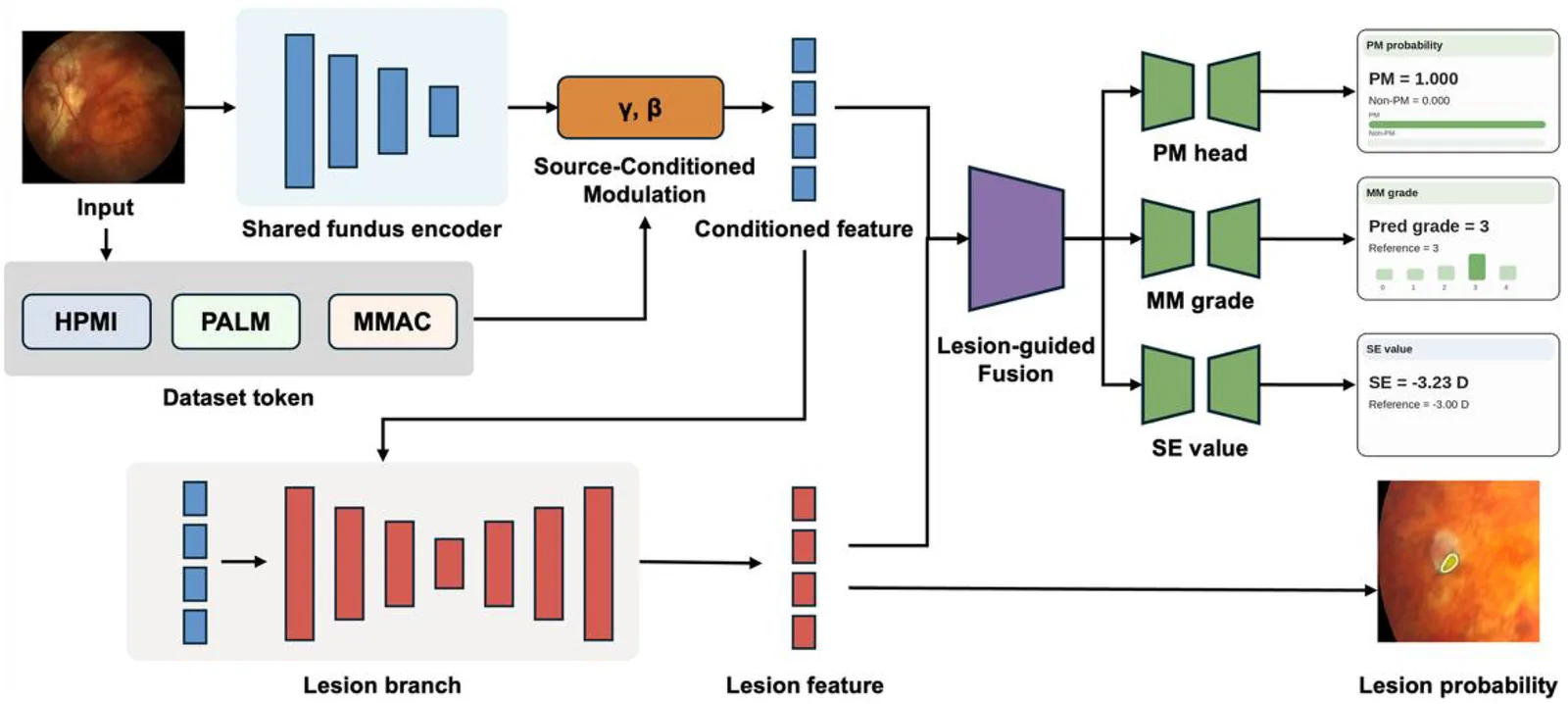
Architecture of myoSpectrumNet. An input fundus image is first encoded by a shared fundus encoder. A source-conditioned modulation module adapts the intermediate representation according to dataset source, after which the conditioned feature is processed along two paths: a lesion-aware branch that produces lesion probability maps and lesion cues, and a lesion-guided fusion module that combines lesion cues with the shared representation before PM, MM, and SE prediction heads. Final outputs include PM probability, MM grade, SE estimate, and lesion-aware interpretation maps.

To reduce appearance and protocol mismatch across HPMI, PALM, and MMAC, we introduced a lightweight feature-wise linear modulation (FiLM)-style dataset-aware adapter, denoted in the architecture as Source-Conditioned Modulation. The dataset indicator was encoded as a source index and mapped through a small learnable embedding/projection layer to generate source-specific channel-wise scaling and shifting vectors. For a dataset indicator, the shared representation was modulated as shown in Equation 2:

$$\tilde{h}=\gamma_{s}⊙h+\beta_{s}$$ (2)

More explicitly, the source index s was first mapped to a learnable embedding, and a small projection generated the source-specific FiLM parameters through the source-only transformation in Equation 3:

$$e_{s}=E_{src}(s),\;[\gamma_{s},\beta_{s}]\;=P_{s}(e_{s})$$ (3)

The vectors gamma_s and beta_s were matched to the channel dimension of the encoder representation and applied feature-wise as shown above. In implementation, gamma_s and beta_s were derived only from dataset identity and not from disease labels, class labels, SE values, or lesion annotations at inference. Thus, the module functioned as a source adapter for acquisition and protocol heterogeneity rather than as a shortcut to task labels. This mechanism allowed the model to preserve a common visual backbone while adapting intermediate representations to source-specific distributional differences.

The conditioned feature was then used in two paths. First, task-specific heads produced outputs for PM screening, MM grading, and SE estimation. Second, a lesion-aware branch generated lesion probability maps and extracted lesion cues from supervision available in MMAC and auxiliary anatomy/lesion signals available in PALM. The branch used a lightweight decoder to upsample encoder features and produce dense lesion logits; the resulting probability maps were also pooled/projected to form a compact lesion representation. This lesion representation was combined with the source-conditioned global feature through lesion-guided fusion, as defined in Equation 4:

$$z=F\left(\tilde{h},l\right)$$ (4)

Operationally, the lesion decoder produced dense probability maps and compact lesion features, and lesion-guided fusion was implemented with a residual gate, as specified in Equation 5:

$$m=\sigma \left(D\left(\tilde{h}\right)\right),g=\sigma \left(W_{g}\left[\tilde{h};l\right]+b_{g}\right),z=\tilde{h}+g⊙P_{l}\left(l\right)$$ (5)

Here D denotes the lesion decoder, l denotes the compact lesion representation obtained by pooling and projecting decoder features, W_g and b_g parameterize the residual gate, and P_l projects lesion information to the same feature dimension as the source-conditioned global feature. The resulting task-aware feature z was passed to the PM, MM, and SE heads. In practical terms, the lesion pathway supplied explicit spatial evidence, while the learned gate controlled how strongly lesion information updated the shared feature for each image. This design enabled the network to incorporate lesion evidence explicitly, rather than relying solely on implicit attention patterns, thereby improving lesion-aware interpretation while preserving the unified nature of the framework.

We use the term lesion-aware interpretation consistently to denote the model’s supportive lesion probability maps, lesion overlays, and lesion-conditioned feature contribution. These outputs are not intended to represent an autonomous clinical lesion diagnosis; rather, they provide visual evidence that may help users understand which pathologic regions contributed to screening or stratification outputs.

### 2.4 Training strategy and objective functions

Training was performed under a masked multi-task setting because not every image had labels for every task. For each sample, only the losses corresponding to available annotations were activated during backpropagation. The overall training objective was defined in Equation 6:

$$L\;=m_{PM}\lambda_{PM}L_{PM}+m_{MM}\lambda_{MM}L_{MM}+m_{SE}\lambda_{SE}L_{SE}+m_{les}\lambda_{les}L_{les}$$ (6)

where the task-availability mask indicated whether the current sample contained supervision for a given task, and the corresponding loss weight controlled the contribution of that task. The PM loss was a binary classification loss, the MM loss was a multi-class grading loss, the SE loss was a regression loss, and the lesion loss was a dense segmentation loss over available lesion masks. In the objective above, *λ*PM, *λ*MM, *λ*SE, and *λ*les denote the four task-loss coefficients. Of the three named hyperparameters reported for the retained checkpoints, only the segmentation weight maps directly to this objective, as *λ*les = 0.30. The concept-fusion coefficient (0.15) and joint-fusion coefficient (0.20 in the balanced checkpoint and 0.25 in the interpretation checkpoint) controlled the lesion-guided fusion path outside the objective, whereas *λ*PM, *λ*MM, and *λ*SE were held fixed across the compared checkpoints.

The main model used a batch size of 12, learning rate of 3 × 10⁻⁴, weight decay of 1 × 10⁻⁴, maximum of 18 epochs, and early-stopping patience of 5 epochs. The adapter dimension was 64 and the decoder channel width was 96. Model selection used validation performance only, with test data excluded from checkpoint selection. The overall monitor used for checkpoint selection and in Table 3 was the unweighted arithmetic mean of HPMI PM AUC, PALM PM AUC, and MM-grading QWK; SE MAE and lesion Dice were reported separately and were not included in this monitor.

**Table 3:** Ablation study of the proposed framework. Overall monitor is the unweighted arithmetic mean of HPMI PM AUC, PALM PM AUC, and MM-grading QWK. The table summarizes the effects of partial-label training, the dataset-aware adapter, lesion-aware design, and refinement variants. The retained final model achieved the best overall monitor, whereas the tested consistency variant did not improve core performance.

Method	Overall monitor	PM on HPMI AUC	PM on PALM AUC	MM grading QWK	SE regression MAE	Lesion Dice
Naive MTL	0.957	0.987	0.995	0.888	1.201	-
Partial-label MTL	0.949	0.987	0.991	0.870	1.093	-
Adapter baseline	0.96	0.985	0.995	0.900	1.502	-
Adapter + consistency	0.938	0.982	0.993	0.839	1.474	-
Adapter + lesion auxiliary	0.94	0.988	0.997	0.836	1.153	0.343
Adapter + lesion concept	0.959	0.987	0.995	0.896	1.261	0.325
MyoSpectrumNet	0.962	0.985	0.993	0.907	1.259	0.411
Interpretation checkpoint	0.952	0.988	0.995	0.873	1.095	0.472

The primary PM and MM comparative baselines were independently trained task-specific ConvNeXt-T models. HPMI and PALM PM classifiers used 384 × 384 inputs, batch sizes of 24 and 12, respectively, a learning rate of 3 × 10⁻⁴, weight decay of 1 × 10⁻⁴, maximum of 20 epochs, and patience of 5 on the HPMI 2,406/802/803 and PALM 400/400/400 train/validation/test splits. MM grading used a five-class ConvNeXt-T model on the MMAC 1,143/248 task-specific train/validation partition with 384 × 384 inputs, batch size 16, and the same learning rate, weight decay, epoch limit, and patience.

For the additional SE comparator, an ImageNet-1K-pretrained ConvNeXt-T with a scalar regression head was trained on the official 992-record MMAC SE training partition and evaluated once on the untouched 205-record validation partition. Within the training partition, a target-quantile-stratified 80/20 holdout selected the learning rate from {1 × 10⁻⁴, 3 × 10⁻⁴} and the stopping epoch (maximum 20; patience 5); AdamW, weight decay 1 × 10⁻⁴, dropout 0.1, Smooth L1 loss (beta 0.5), and effective batch size 16 were fixed in advance. The selected learning rate was 1 × 10⁻⁴ and epoch 18. Target mean and SD were estimated only from the relevant training records, and predictions were inverse-transformed as prediction_D = prediction_z × SD_D + mean_D before any metric or figure was generated. The official validation labels were not used for normalization, tuning, or checkpoint selection.

For the additional lesion comparator, three ImageNet-1K-pretrained DeepLabV3-ResNet50 binary segmenters were trained independently for CNV, Fuchs spot, and lacquer cracks on the official 149 MMAC lesion training records and evaluated on the untouched 32-record validation partition. Lesion-area-stratified 80/20 internal holdouts selected the learning rate from {1 × 10⁻⁴, 3 × 10⁻⁴}, stopping epoch (maximum 40; patience 8), and probability threshold from {0.3, 0.4, 0.5, 0.6, 0.7}. AdamW, weight decay 1 × 10⁻⁴, a 0.5 focal-loss plus 0.5 soft-Dice objective, 384 × 384 inputs, and effective batch size 8 were fixed. No TTA was used in the primary comparison.

All primary head-to-head rows used single-view inference for both the task-specific comparator and MyoSpectrumNet. No TTA was applied to either model in these rows. Four-view TTA appears only in explicitly labeled within-MyoSpectrumNet sensitivity conditions. The SE and lesion comparisons used the identical official MMAC partitions and 384 × 384 evaluation grid. For the original MyoSpectrumNet checkpoints, auxiliary-task outputs were available for this revision only as aggregate summaries rather than record-level predictions. Cross-model differences are therefore arithmetic only, with no paired confidence interval or between-model *P* value, and are interpreted descriptively.

### 2.5 Evaluation protocol, calibration, and statistical analysis

MMAC provides official training and validation partitions for MM grading, SE estimation, and lesion segmentation but no separate test partition. Accordingly, MyoSpectrumNet metrics for these outputs were reported on the same validation partitions used during model selection, whereas each task-specific comparator selected hyperparameters only within its training records before one evaluation on the official validation partition. Matching the split, evaluation grid, and inference setting improves comparability but does not make these auxiliary analyses independent test-set comparisons. Evaluation was otherwise performed in the two source settings described above and in the two task-specific auxiliary-comparator experiments. PM screening was assessed by ROC AUC, accuracy, F1 score, sensitivity, and specificity; MM grading by accuracy, macro-F1, grade MAE, and QWK; SE estimation in native diopters by MAE, RMSE, R², Pearson r, and Spearman rho; and lesion segmentation by per-record Dice. For the SE and lesion baselines, 95% percentile-bootstrap confidence intervals used 10,000 resamples of the official MMAC validation records. Single-view point estimates were used for the primary comparisons; interpretation of the unpaired auxiliary gaps follows the disclosure above.

To assess probability reliability in screening-oriented deployment, we further performed *post-hoc* temperature scaling on PM screening outputs. Given a logit vector z_i_, the calibrated probability was defined by Equation 7:

$$\begin{array}{c} p_{i}=\mathrm{softmax}\left(\frac{z_{i}}{T}\right) \end{array}$$ (7)

where T is a learned temperature estimated on validation predictions and then applied only at inference. Calibration was summarized using expected calibration error (ECE), Brier score, and negative log-likelihood (NLL). Cross-model comparisons use protocol-matched single-view point estimates, whereas four-view TTA and temperature scaling are evaluated descriptively within the same checkpoint. The manuscript therefore makes no claim of statistically significant cross-model superiority or of an architecture-only calibration advantage.

### 2.6 Computational environment, implementation, and reproducibility details

All model development and image analysis were conducted in Python 3.10. Deep learning experiments were implemented using PyTorch 2.1.0 with CUDA 11.8 acceleration. Data processing and numerical analysis were performed using NumPy 1.26.4, pandas 2.1.4, SciPy 1.11.4, and scikit-learn 1.3.2. Image preprocessing and visualization were performed using OpenCV 4.8.1 and Matplotlib 3.8.2. Statistical analyses and figure organization were conducted in Python, and all experiments were run in a single CUDA-enabled Linux GPU environment.

The additional SE and lesion comparator experiments were executed in Python 3.10.16 using PyTorch 2.0.0, torchvision 0.15.0, CUDA 11.7, NumPy 1.25.2, pandas 2.0.3, SciPy 1.11.1, and scikit-learn 1.3.0 on one NVIDIA GeForce RTX 4,060 GPU. Official torchvision ImageNet-1 K weights were used. All auxiliary-comparator training runs used fixed seed 20,260,811, and bootstrap resampling used seed 20,260,812. Dataset archives were verified against the publisher-provided MD5 checksums before extraction.

For computational profiling, MyoSpectrumNet used 384 × 384 inputs, batch size 1, and a single 24-GB NVIDIA CUDA-enabled GPU; CPU preprocessing, image decoding, disk input/output, and visualization were excluded. The model contained approximately 31 million trainable parameters. Mean latency was approximately 25 ms for single-view inference and 95 ms for four-view inference. These values describe model-level latency in the reported environment and should be re-evaluated in any intended clinical workflow.

## 3 Results

### 3.1 Overall performance of MyoSpectrumNet

Under matched-split single-view inference, the task-specific baselines and MyoSpectrumNet yielded HPMI PM AUCs of 0.987 and 0.985, PALM PM AUCs of 0.996 and 0.993, and MM-grading QWK values of 0.907 and 0.907, respectively. The SE baseline achieved MAE 1.006 D (95% CI 0.873–1.147), RMSE 1.413 D, and Pearson r 0.801, compared descriptively with the aggregate MyoSpectrumNet single-view MAE of 1.259 D. The standalone lesion baseline achieved overall Dice 0.397 (95% CI 0.324–0.468), compared descriptively with 0.472 for the MyoSpectrumNet interpretation checkpoint under single-view inference. Because the original MyoSpectrumNet auxiliary values were retained only as aggregate summaries, the SE and lesion gaps are arithmetic rather than inferential comparisons. Four-view TTA remained a within-MyoSpectrumNet sensitivity condition (Figure 5; Table 4; Table 5, and Supplementary Tables S2A-S3B).

**Figure 5:**
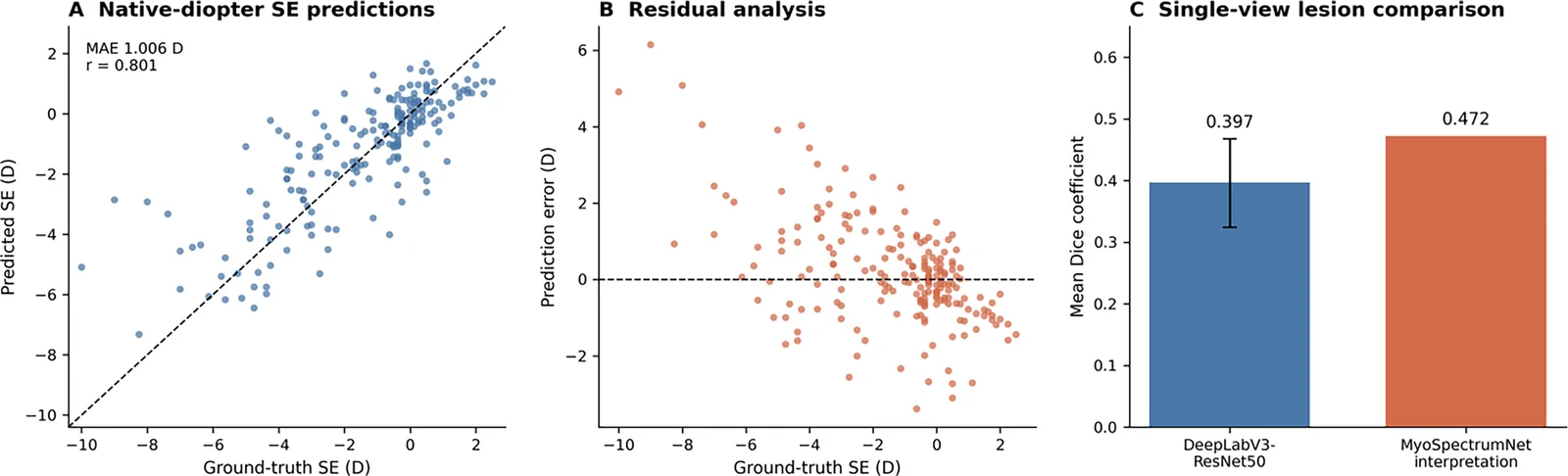
Auxiliary-task comparator analyses. **(A)** Predictions from the single-task ConvNeXt-T SE regressor in native diopters. **(B)** Residuals against ground-truth SE. **(C)** Mean Dice for the standalone DeepLabV3-ResNet50 comparator and the MyoSpectrumNet interpretation checkpoint under single-view inference on the identical MMAC 149/32 lesion split and 384 × 384 grid. The error bar is the standalone baseline’s 95% bootstrap CI; the MyoSpectrumNet point estimate is a legacy aggregate summary. Because this interval does not quantify the between-model difference, the panel is descriptive and does not establish model ordering.

**Table 4:** Matched-split descriptive performance summary. Every primary cross-model row uses single-view inference on the same task-specific split. Values in parentheses for the auxiliary-task baselines are 95% percentile-bootstrap confidence intervals. MyoSpectrumNet auxiliary values are unpaired aggregate summaries; interpretation follows Section 2.5. Four-view TTA rows are within-MyoSpectrumNet sensitivity conditions and are not substituted into the primary single-view comparisons.

Method	PM on HPMI AUC	PM on PALM AUC	MM grading QWK	SE MAE (D)	Lesion Dice
HPMI PM baseline (ConvNeXt-T; single-view)	0.987	—	—	—	—
PALM PM baseline (ConvNeXt-T; single-view)	—	0.996	—	—	—
MM baseline (ConvNeXt-T; single-view)	—	—	0.907	—	—
SE baseline (ConvNeXt-T; single-view)	—	—	—	1.006 (0.873–1.147)	—
Lesion baseline (DeepLabV3-ResNet50; single-view)	—	—	—	—	0.397 (0.324–0.468)
MyoSpectrumNet (single-view)	0.985	0.993	0.907	1.259	0.411
MyoSpectrumNet + four-view TTA	0.989	0.994	0.913	1.264	0.414
Interpretation checkpoint (single-view)	—	—	—	—	0.472
Interpretation checkpoint + four-view TTA	—	—	—	—	0.480

**Table 5:** Matched-split single-view task comparisons and within-model TTA sensitivity. Delta is MyoSpectrumNet minus the task-specific baseline; negative values favor MyoSpectrumNet for MAE, whereas positive values favor it for AUC, QWK, and Dice. Baseline confidence intervals are shown where calculated. The SE and lesion deltas are unpaired arithmetic differences without confidence intervals or paired *P* values; interpretation follows Section 2.5.

Task	Metric	Task-specific baseline (single-view)	MyoSpectrumNet (single-view)	Single-view delta	MyoSpectrumNet + four-view TTA (change vs. single-view)
PM on HPMI	AUC	0.987	0.985	−0.002	0.989 (+0.004)
PM on PALM	AUC	0.996	0.993	−0.003	0.994 (+0.001)
MM grading	QWK	0.907	0.907	0.000	0.913 (+0.006)
SE regression	MAE (D)	1.006 (0.873–1.147)	1.259	+0.253	1.264 (+0.005)
Lesion segmentation (interpretation checkpoint)	Mean Dice	0.397 (0.324–0.468)	0.472	+0.075	0.480 (+0.008)

### 3.2 PM-only cross-dataset transfer analysis

Figures 3A–C and Table 2 report PM-only source-to-target controls rather than leave-one-dataset-out evaluation of the full MyoSpectrumNet checkpoint. A source-specific PM classifier was trained and selected on the source dataset, locked, and applied to the target dataset without target labels or fine-tuning; the source-conditioned multi-task adapter was not used. HPMI-to-PALM transfer yielded an AUC of 0.993 and accuracy of 0.970, whereas PALM-to-HPMI transfer yielded an AUC of 0.969 and accuracy of 0.923. Backbone replacement did not remove this asymmetry: ConvNeXt-T provided the most balanced transfer behavior, whereas ViT-B/16 was less robust. These controls characterize PM-screening transportability between two public sources and do not establish generalization of the full four-output model to a previously unseen dataset or prospective cohort. No MM-grading, SE-estimation, or lesion-aware output was evaluated in an unseen independent cohort.

### 3.3 Ablation analysis and model selection

Ablation analysis supported retention of both the dataset-aware adapter and the lesion-aware design in the final framework. Relative to naive multi-task learning, the adapter baseline improved the overall validation monitor and stabilized MM grading, whereas the tested consistency variant reduced overall performance and was excluded from the final configuration. The ablation results also showed that single metrics could be optimized at the expense of other clinically relevant outputs. For example, partial-label MTL and naive MTL yielded lower SE MAE values than the retained model, but they simultaneously reduced MM grading performance and the overall monitor, while lesion-oriented variants improved lesion Dice at the cost of MM grading or overall balance. The retained MyoSpectrumNet configuration was therefore selected as the best balanced diagnostic checkpoint across PM screening, MM grading, SE estimation, and lesion-aware interpretation, achieving the highest overall monitor among the retained variants together with a lesion Dice of 0.411. These trends are shown in Figure 6 and Table 3, and the optimization trajectories in Supplementary Figure S3 further indicate that the retained configuration was associated with more favorable training dynamics.

**Figure 6:**
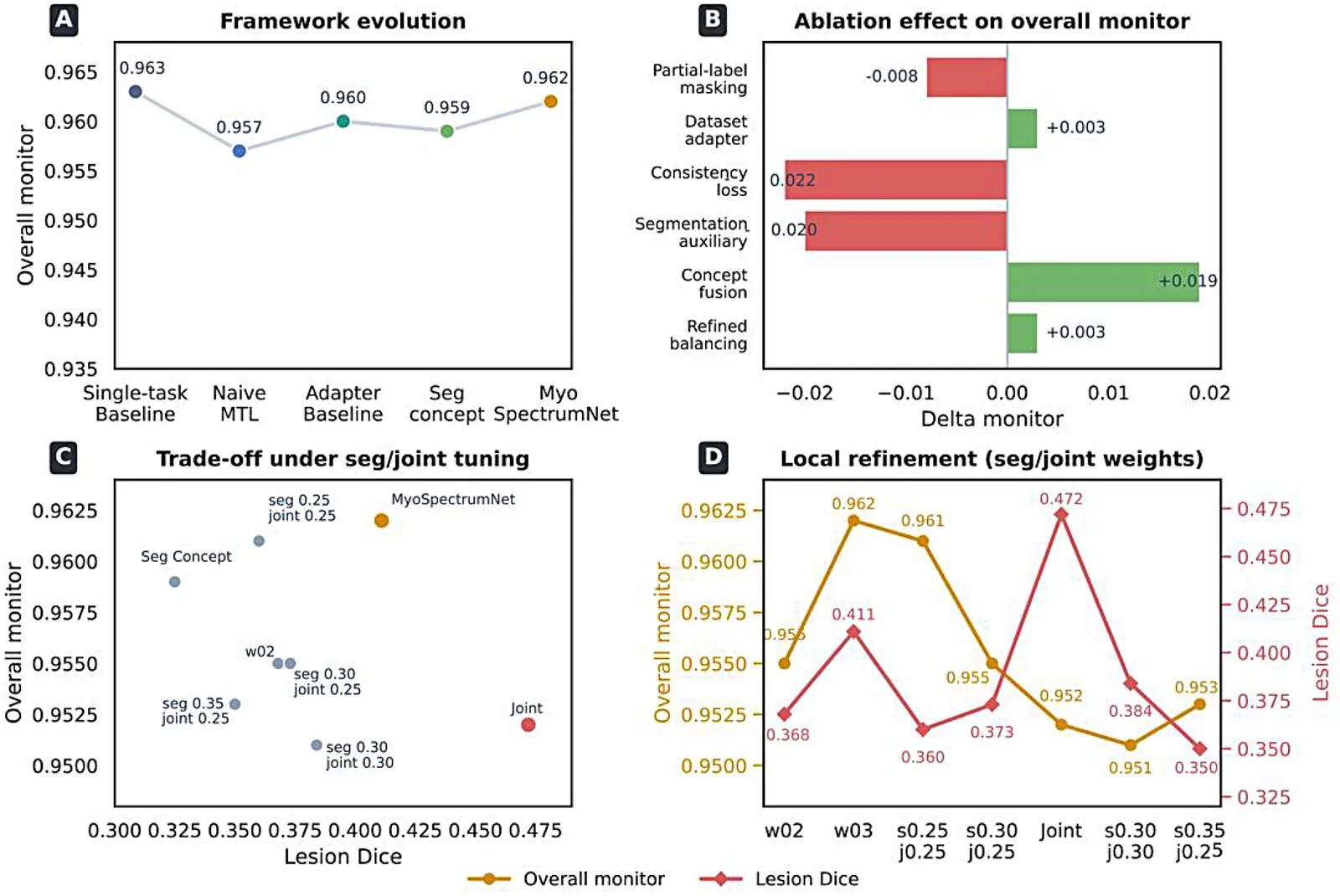
Ablation and trade-off analysis. **(A)** Performance evolution from single-task learning to the retained final framework. **(B)** Incremental changes associated with key design choices. **(C)** Trade-off between overall performance and lesion-aware interpretation quality under different lesion and joint-weighting strategies. **(D)** Local refinement around the retained operating region. The dataset-aware adapter and lesion-aware design were retained in the final framework, whereas the tested consistency variant was not.

The dual-checkpoint strategy was also supported by the ablation results. Whereas MyoSpectrumNet was selected as the main framework for balanced multi-task reporting, the interpretation checkpoint achieved the highest lesion Dice (0.472 without TTA and 0.480 with TTA) at the cost of lower MM grading performance, supporting its use for lesion-focused qualitative analysis rather than as the primary diagnostic checkpoint. This trade-off is clinically relevant because lesion interpretability, refractive-error estimation, and diagnostic severity grading are related but not fully aligned operating targets in heterogeneous fundus learning.

### 3.4 Lesion-aware interpretation

The independently trained DeepLabV3-ResNet50 comparator achieved an overall single-view Dice of 0.397 (95% CI 0.324–0.468) on the official 32-record MMAC lesion validation partition. Lesion-specific baseline Dice values were 0.262 for CNV, 0.420 for Fuchs spot, and 0.451 for lacquer cracks. On the identical split and 384 × 384 grid, the balanced and interpretation MyoSpectrumNet checkpoints had aggregate single-view Dice values of 0.411 and 0.472, corresponding to arithmetic differences of 0.014 and 0.075 from the baseline. These legacy candidate summaries could not be paired with the per-record baseline outputs, so no confidence intervals for the differences or paired *P* values were calculated. Given the 32-record sample, the numerical gaps remain descriptive. The four-view interpretation-checkpoint value of 0.480 is a secondary within-model TTA result (Figure 5C and Supplementary Table S3B).

Qualitative examples illustrated both the usefulness and the limitations of lesion-aware visual evidence. Figure 7 shows representative successful and challenging cases with reference and predicted contours together with activation maps. The examples indicate that the lesion-focused checkpoint can localize clinically relevant regions, while failures remain evident for small, low-contrast, or structurally complex lesions. These maps should therefore be interpreted as explanatory support for screening and triage rather than definitive lesion boundaries.

**Figure 7:**
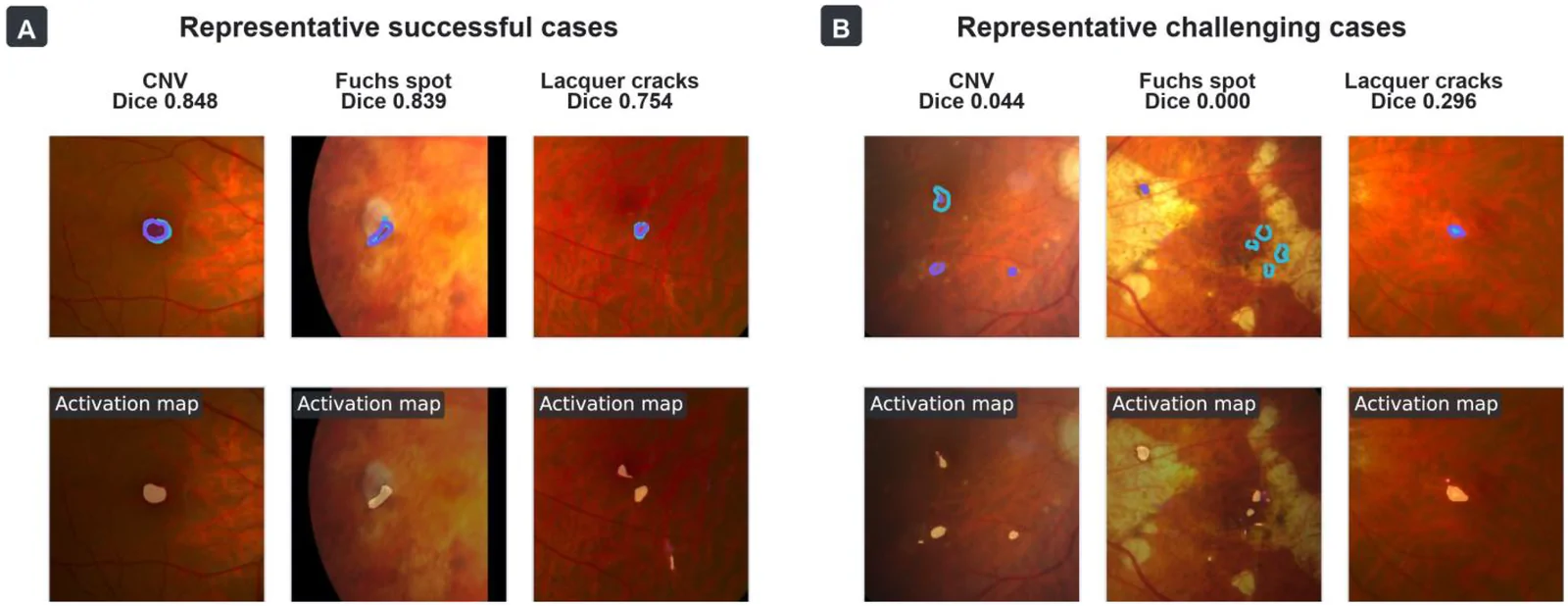
Qualitative lesion-aware interpretation. **(A)** Representative successful lesion-localization cases. **(B)** Representative challenging cases. Cyan contours indicate reference boundaries, purple contours indicate predictions, and activation maps show the model’s lesion-aware visual evidence. The examples are qualitative and do not establish superiority over an optimized standalone segmenter.

### 3.5 Clinical stratification and calibration

Subgroup analyses indicated generally stable PM-screening behavior across PALM devices and shooting centers, with AUCs ranging from 0.988 to 0.995 (Supplementary Table S4A). MM grading declined in the highest severity bin, where accuracy was 0.680 and grade MAE was 0.440. The row-normalized grade-4 sensitivity was 1.00 for the task-specific baseline and 0.77 for MyoSpectrumNet with four-view TTA; grades 3–4 represented only 25 records combined, so the highest-grade estimate is unstable. Auxiliary SE error also increased in the most myopic subgroup. These descriptive analyses identify difficult subgroups for future validation; they are not evidence of prospective clinical generalizability.

Within the four-view MyoSpectrumNet outputs, temperature scaling reduced ECE from 0.028 to 0.021 on HPMI and from 0.023 to 0.020 on PALM. Brier score and NLL changed from 0.030 to 0.028 and from 0.124 to 0.100 on HPMI, and from 0.024 to 0.023 and from 0.111 to 0.090 on PALM. This is a within-checkpoint *post-hoc* calibration sensitivity analysis and does not establish an architectural calibration advantage (Figure 8, Table 6, and Supplementary Tables S4B-S4C).

**Figure 8:**
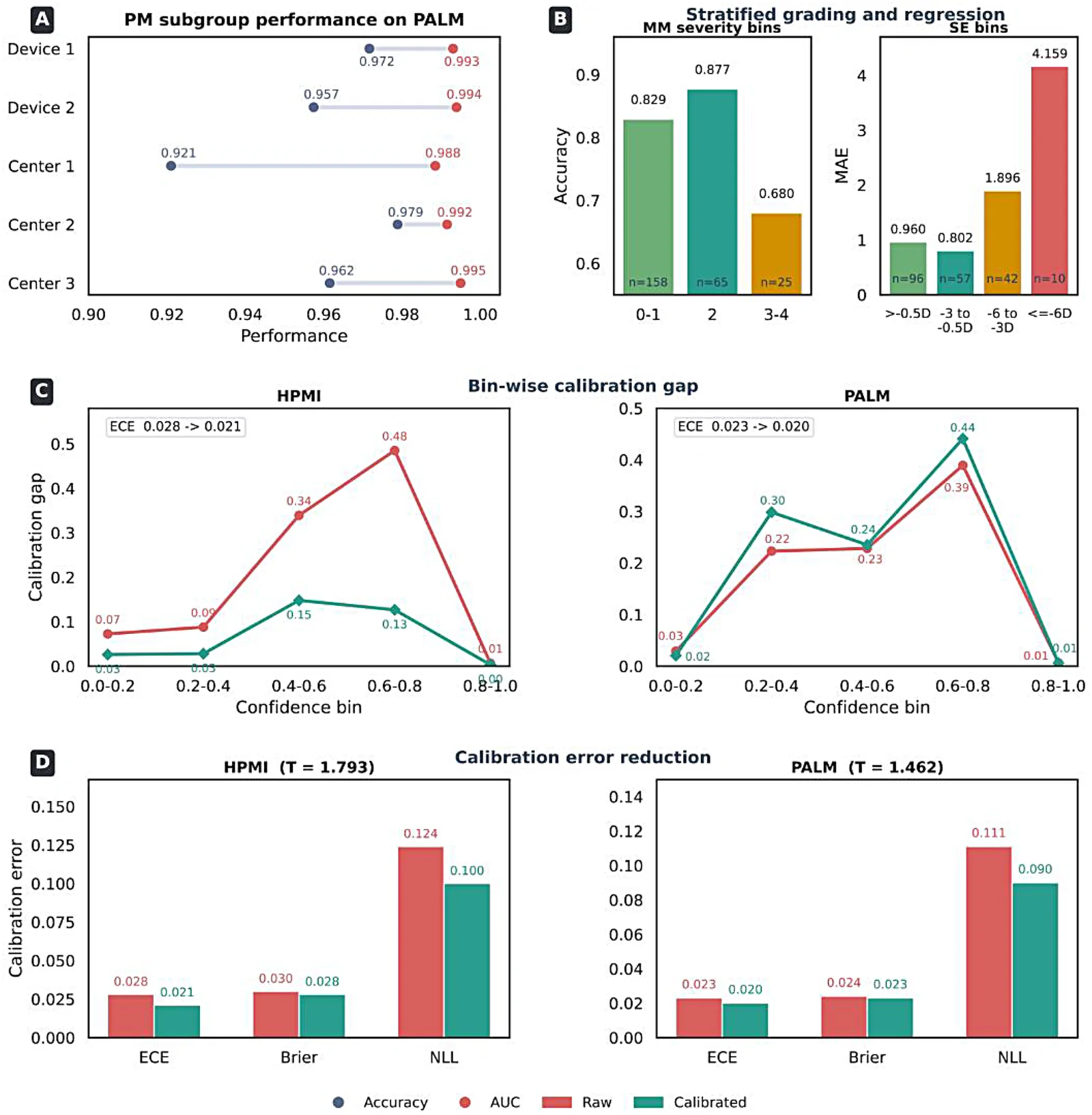
Clinical stratification and calibration analysis. **(A)** PM subgroup performance on PALM. **(B)** MM grading and auxiliary SE estimation across clinically relevant subgroups. **(C)** Calibration-gap trends for raw and temperature-scaled four-view MyoSpectrumNet outputs on HPMI and PALM. **(D)** Within-checkpoint changes in ECE, Brier score, and NLL. The calibration panels do not compare architectures or inference protocols.

**Table 6:** Within-model calibration and threshold-oriented screening analysis. Raw and temperature-scaled metrics are shown for the same four-view MyoSpectrumNet checkpoint. Lower ECE, Brier score, and NLL indicate better probability reliability. These results do not compare MyoSpectrumNet with task-specific baselines or isolate architecture from inference protocol.

Dataset	Output	ECE	Brier	NLL	ECE change after scaling	Threshold ≥0.90 sensitivity	Threshold ≥0.95 sensitivity
HPMI	Four-view raw	0.028	0.030	0.124	Reference	0.937	0.734
HPMI	Four-view temperature-scaled	0.021	0.028	0.100	−0.007	0.819	0.650
PALM	Four-view raw	0.023	0.024	0.111	Reference	0.333	0.333
PALM	Four-view temperature-scaled	0.020	0.023	0.090	−0.003	0.397	0.397

For PALM, the same empirical threshold satisfied both sensitivity targets in the observed score distribution; the identical values therefore reflect the discrete empirical operating points rather than separate threshold tuning.

### 3.6 Failure analysis and statistical support

Representative failure cases revealed three recurring error patterns: boundary ambiguity in PM screening, underestimation of MM severity in advanced disease, and lesion segmentation failure in small or low-contrast lesions. As illustrated in Figure 9, false-positive and false-negative PM cases frequently involved fundus appearances near the decision boundary or substantial morphological overlap with non-PM eyes. MM grading errors were more often observed in severe structural distortion, whereas lesion segmentation failures were concentrated in subtle lesions, complex backgrounds, or visually fragmented lesion regions. Additional examples in Supplementary Figure S4 confirmed that these residual errors were systematic rather than isolated.

**Figure 9:**
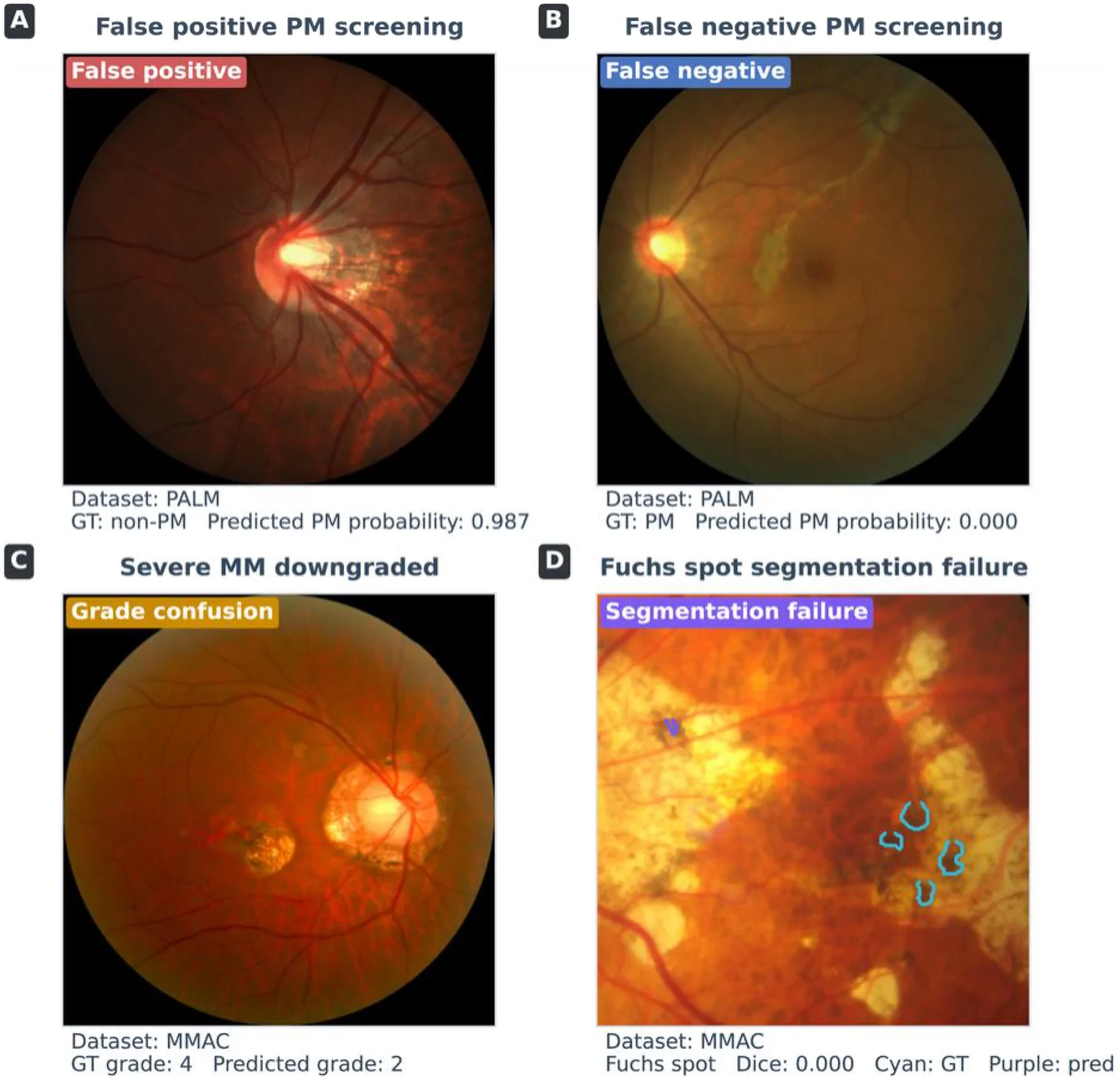
Representative failure cases. Panels show false-positive and false-negative PM screening cases, MM severity underestimation, and lesion-aware interpretation failures. These cases highlight residual challenges related to boundary ambiguity, severe morphological variation, and difficulty in detecting small or low-contrast lesions.

The primary matched-split comparisons are the single-view point estimates in Figure 10A, Figure 10, Table 5, and Supplementary Table S3A. Figure 10C,D retains the HPMI and PALM ROC curves and Figure 10E the MM confusion matrices. Figure 5 presents native-diopter SE predictions together with the single-view lesion comparator. Four-view TTA and temperature scaling remain explicitly labeled within-model sensitivity analyses.

**Figure 10:**
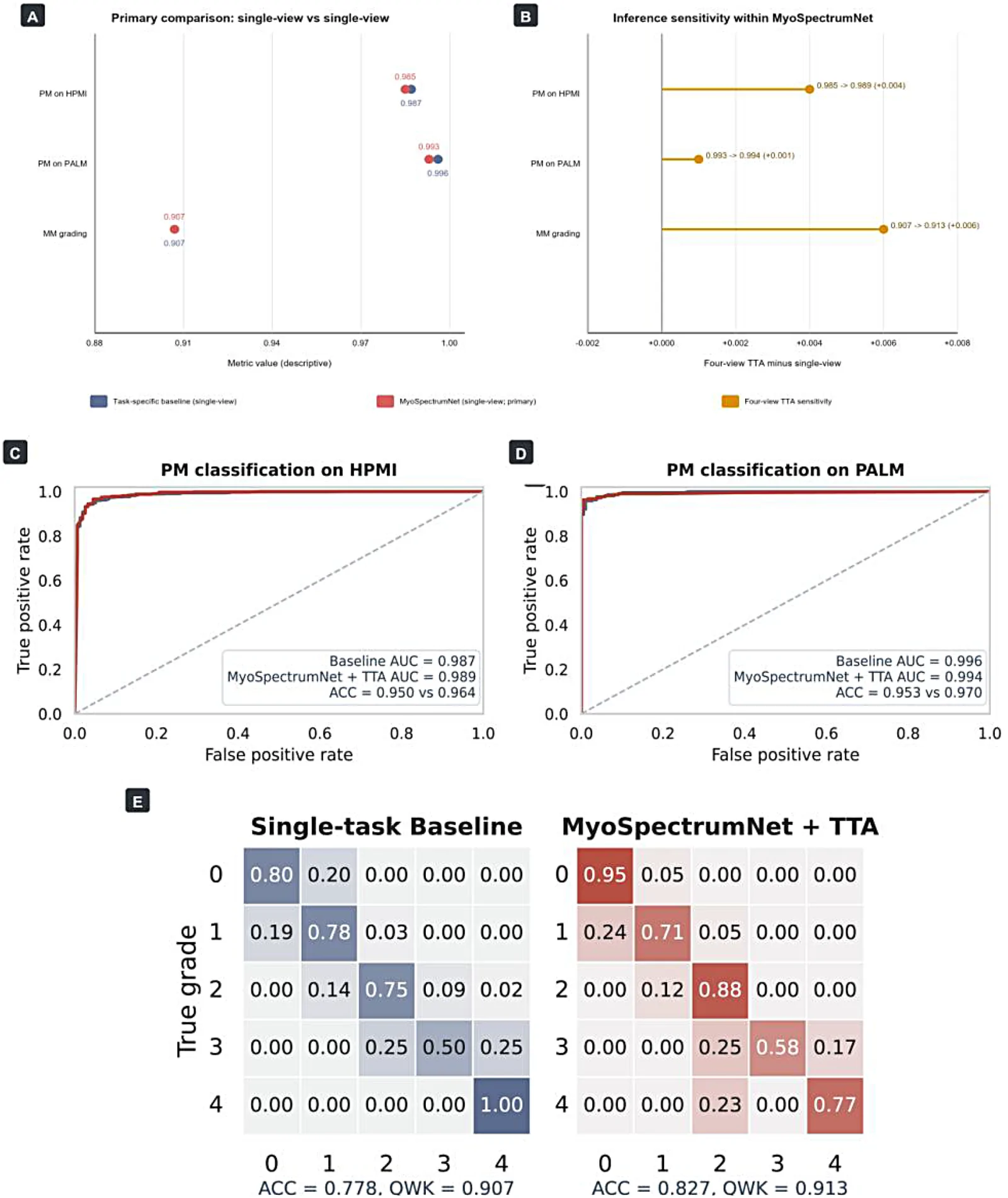
Main screening and grading performance. **(A)** Matched-split single-view point estimates for HPMI PM screening, PALM PM screening, and MM grading. **(B)** Within-MyoSpectrumNet changes from single-view inference to four-view TTA. **(C,D)** ROC curves for HPMI and PALM operational outputs. **(E)** Row-normalized MM-grading confusion matrices. The task-specific SE analysis is presented in Figure 10A,B.

## 4 Discussion

In this study, MyoSpectrumNet returned PM screening, MM grading, auxiliary SE, and lesion-aware visual evidence from one shared representation. Under protocol-matched single-view inference, PM AUC and MM QWK point estimates were similar to those of the corresponding task-specific models. The rationale for joint learning is therefore integration of complementary partial labels, shared computation, and consistent multi-output reporting rather than an expectation of improvement on every task. Four-view TTA and temperature scaling are reported only as within-model sensitivity analyses and are not evidence of architectural superiority.

The rationale for this design is rooted in the clinical and data-related complexity of PM itself. PM is defined not simply by refractive error, but by structural abnormalities of the posterior segment, and its evaluation commonly involves both global severity judgment and lesion-specific assessment (3–6). In parallel, currently available public datasets do not provide identical supervision: HPMI mainly supports screening, PALM adds anatomy- and lesion-related cues, and MMAC extends supervision to MM grading, SE estimation, and plus-lesion analysis (23–26). As a result, PM intelligence cannot be optimally framed as a conventional single-dataset classification benchmark. Instead, it is better understood as a heterogeneous multi-source learning problem in which complementary but partially aligned annotations must be integrated without forcing artificial label uniformity. This was the conceptual basis for our study design.

The PM-only source-to-target controls emphasize the effect of public-dataset source shift. These experiments used conventional source-specific PM classifiers rather than the source-conditioned multi-task checkpoint. Transfer was asymmetric, with lower performance in the PALM-to-HPMI direction, and backbone substitution did not remove the gap. Accordingly, the results support only a narrow PM-screening transportability claim. They do not provide external validation of the complete framework: MM grading, SE estimation, and lesion-aware outputs were never tested in an unseen independent cohort. Until a leave-one-source-out or prospective multi-output evaluation is performed, the four-output checkpoint should be regarded as evaluated only on held-out partitions from its contributing sources, not as generally validated across institutions or acquisition domains.

Lesion-aware output adds spatial evidence to an otherwise label-based workflow. In the matched-split single-view comparison, the DeepLabV3-ResNet50 baseline achieved Dice 0.397 (95% CI 0.324–0.468), while the balanced main checkpoint and lesion-focused interpretation checkpoint achieved aggregate Dice values of 0.411 and 0.472, respectively. The main-checkpoint value lies within the baseline interval, whereas the interpretation-checkpoint value is only 0.004 above its upper limit. Because that interval describes uncertainty in the baseline estimate alone rather than uncertainty in a paired between-model difference, it does not resolve the model ordering. With only 32 validation records, lesion-specific counts are sparse and the point estimates are sensitive to case mix and individual difficult cases. Independent lesion-level validation with retained record-level predictions is therefore required.

The task-specific SE comparison used an independently trained ConvNeXt-T regressor on the official 992/205 MMAC split. The training targets had mean -1.598 D and population SD 2.555 D. On the official validation partition, the baseline achieved MAE 1.006 D (95% CI 0.873–1.147) and Pearson r 0.801 (95% CI 0.746–0.849), with predictions spanning -7.32 to 1.67 D rather than clustering near the training mean. MyoSpectrumNet’s aggregate single-view MAE was 1.259 D. The integrated model therefore did not outperform the task-specific regressor on this outcome (Figures 5A,B). The unpaired 0.253 D arithmetic gap is descriptive and should not be interpreted as a statistically tested between-model effect.

Several limitations should be acknowledged. First and most importantly, external validation of the complete multi-output framework was not performed. The all-source checkpoint was evaluated only on held-out partitions from known sources, and the source-to-target controls assessed PM screening alone. Thus, unseen-source generalizability of MM grading, SE estimation, and lesion-aware outputs remains unknown, and the four-output checkpoint should not be described as externally validated. Second, the lesion validation partition contained only 32 records. This small sample produces substantial sampling uncertainty, limits lesion-specific inference, and makes the observed numerical gaps sensitive to case mix. Third, only legacy aggregate MyoSpectrumNet SE and lesion estimates were available for this revision, and the original checkpoint files and record-level auxiliary predictions were unavailable; paired differences and their uncertainty could therefore not be estimated. Fourth, MMAC provides no separate test partition, so the unified-model MM, SE, and lesion results are selection-set estimates rather than independent test results. Fifth, the auxiliary comparators used transparent but limited hyperparameter grids and one fixed training seed; broader architecture searches or repeated training could change the point estimates. Sixth, the public datasets differ in population, acquisition, annotation, and task definition, and patient-level overlap could not be directly audited. Finally, grades 3–4 contributed only 25 MM-grading records. Prospective multi-center, unseen-source, independently tested, and fully paired evaluations of all outputs remain necessary.

## 5 Conclusion

In conclusion, MyoSpectrumNet provides a unified research framework for PM screening, MM grading, auxiliary SE estimation, and lesion-aware interpretation across complementary public datasets. The task-specific SE and segmentation comparators clarify the expected trade-offs: the supported contribution is integrated multi-output assessment within the included sources rather than improvement of every individual task. The complete multi-output system has not undergone external validation on an unseen dataset. Independent prospective, multi-center, and workflow-based validation of all four outputs is required before clinical deployment.

## Data Availability

The original contributions presented in the study are included in the article/Supplementary Material, further inquiries can be directed to the corresponding author.
