# Supplementary material for: MyoSpectrumNet: a unified deep learning framework for pathologic myopia spectrum assessment using heterogeneous multi-source fundus datasets
Source: Front Med (Lausanne). 2026 Oct 27;13:1881289. doi: 10.3389/fmed.2026.1881289 (PMC13747858; doi:10.3389/fmed.2026.1881289)
Supplement: Supplementary file 1 [file Datasheet7.pdf]

Supplementary Material

MyoSpectrumNet: A Unified Deep Learning Framework for Pathologic Myopia Spectrum Assessment Using Heterogeneous Multi-Source Fundus Datasets

Reporting scope. MMAC provides training and validation partitions but no independent test partition; unified-model MM, SE, and lesion values are therefore validation/selection-set estimates. The task-specific SE and lesion comparators were tuned only within training records and evaluated once on the official validation partitions. Cross-model SE and lesion differences remain descriptive because record-level MyoSpectrumNet auxiliary outputs were unavailable.

Table S1. Detailed split and annotation coverage. Counts are task-specific records. MMAC tasks may share source images, so the task counts must not be summed to infer the number of unique images.

Dataset	Task	Split	Task-specific records	Primary label records	Disc	Atrophy	Detachment	Fovea
HPMI	PM classification	Train	2406	2406	0	0	0	0
HPMI	PM classification	Validation	802	802	0	0	0	0
HPMI	PM classification	Test	803	803	0	0	0	0
PALM	PM classification	Train	400	400	381	311	12	400
PALM	PM classification	Validation	400	400	379	271	6	400
PALM	PM classification	Test	400	400	384	288	6	400
MMAC	MM grading	Train	1143	1143	0	0	0	0
MMAC	MM grading	Validation	248	248	0	0	0	0
MMAC	SE regression	Train	992	992	0	0	0	0
MMAC	SE regression	Validation	205	205	0	0	0	0
MMAC	Lesion interpretation	Train	149	149	0	0	0	0
MMAC	Lesion interpretation	Validation	32	32	0	0	0	0

Table S2A. Architecture, partition, fixed numerical settings, and searched ranges for the task-specific baselines, unified-model checkpoints, and PM-only transfer controls. Comparator hyperparameter selection was confined to internal holdouts within the relevant training records; unified-model MMAC values remain validation/selection-set estimates because MMAC provides no separate test partition.

Method or protocol	Architecture	Development/evaluation partition	Input size	Batch size	Learning rate	Weight decay	Maximum epochs	Patience
HPMI single-task PM baseline	ConvNeXt-T	HPMI train/validation/test = 2406/802/803	384 × 384	24	3×10^{-4}	1×10^{-4}	20	5
PALM single-task PM baseline	ConvNeXt-T	PALM official train/validation/test = 400/400/400	384 × 384	12	3×10^{-4}	1×10^{-4}	20	5
MMAC single-task MM baseline	ConvNeXt-T	MMAC train/validation = 1143/248	384 × 384	16	3×10^{-4}	1×10^{-4}	20	5
MMAC SE baseline	ImageNet-pretrained ConvNeXt-T; scalar head	Official 992/205; target-quantile 80/20 holdout inside training only	384 × 384	8; accumulation 2 (effective 16)	$\{1 \times 10^{-4}, 3 \times 10^{-4}\}$; selected 1×10^{-4}	1×10^{-4} (fixed)	20; selected 18	5
MMAC lesion baseline	Three ImageNet-pretrained DeepLabV3-ResNet50 binary segmenters	Official 149/32; lesion-area-stratified 80/20 holdout inside training only	384 × 384	2; accumulation 4 (effective 8)	$\{1 \times 10^{-4}, 3 \times 10^{-4}\}$; selected 3×10^{-4} for all lesions	1×10^{-4} (fixed)	40	8
MyoSpectrumNet	ConvNeXt-T encoder; source adapter; task heads; lesion decoder	HPMI, PALM, and MMAC task-specific training partitions; checkpoint selected on contributing validation partitions	384 × 384	12	3×10^{-4}	1×10^{-4}	18	5
MyoSpectrumNet + four-view TTA	Same checkpoint as MyoSpectrumNet	Same held-out validation/test partitions; no retraining	384 × 384	12 during training	3×10^{-4} during training	1×10^{-4} during training	18	5
Interpretation checkpoint + four-view TTA	Same encoder/decoder family; joint-fusion coefficient 0.25	MMAC lesion validation = 32 records; validation/selection-set result	384 × 384	12	3×10^{-4}	1×10^{-4}	18	5
PM-only transfer: HPMI → PALM	ConvNeXt-T primary; additional backbones in Table 3	Train/select HPMI; locked evaluation PALM	384 × 384	24	3×10^{-4}	1×10^{-4}	20	5
PM-only transfer: PALM → HPMI	ConvNeXt-T primary; additional backbones in Table 3	Train/select PALM; locked evaluation HPMI	384 × 384	12	3×10^{-4}	1×10^{-4}	20	5

Table S2B. Model-selection and inference protocol. Primary cross-model comparisons use single-view inference for both models. The SE and lesion comparators were selected only on internal holdouts from the official training records; TTA was not used in their primary comparisons. MMAC has no separate test partition.

Method or protocol	Role	Model selection	Inference
HPMI single-task PM baseline	Task-specific PM classifier	Validation early stopping; test excluded	Single-view; primary comparison
PALM single-task PM baseline	Task-specific PM classifier	Validation early stopping; test excluded	Single-view; primary comparison
MMAC single-task MM baseline	Five-class MM grader	Validation early stopping; validation excluded from training	Single-view; primary comparison
MMAC SE baseline	Task-specific image-only regressor	LR and epoch selected on target-quantile internal holdout; official validation untouched; target scaling fitted on training only	Single-view; inverse-transformed to diopters; no TTA
MMAC lesion baseline	Three task-specific binary segmenters	LR, epoch, and threshold selected on lesion-area internal holdouts; official validation untouched	Single-view; no TTA
MyoSpectrumNet	Primary multi-task checkpoint	Validation monitor (unweighted mean of HPMI PM AUC, PALM PM AUC, and MM-grading QWK); MMAC auxiliary outputs are selection-set estimates	Single-view; primary cross-model comparison
Interpretation checkpoint	Lesion-focused multi-task checkpoint	Validation lesion-quality trade-off; MMAC lesion value is a selection-set estimate	Single-view; primary lesion comparison
MyoSpectrumNet + four-view TTA	Within-model inference sensitivity	Checkpoint fixed before TTA	Four aligned views; outputs averaged
Interpretation checkpoint + four-view TTA	Within-model lesion sensitivity	Checkpoint fixed before TTA	Four aligned views; maps inverse-aligned before averaging
PM-only transfer: HPMI → PALM	Source-to-target PM control	HPMI validation only	Single-view; adapter not used
PM-only transfer: PALM → HPMI	Source-to-target PM control	PALM validation only	Single-view; adapter not used

Table S3A. Matched-split single-view descriptive task comparisons and within-model four-view TTA sensitivity. Delta is MyoSpectrumNet minus the task-specific baseline. The SE and lesion rows use legacy aggregate MyoSpectrumNet summaries and are not paired inferential comparisons.

Task	Metric	Task-specific baseline (single-view)	MyoSpectrumNet (single-view)	Single-view delta	MyoSpectrumNet + four-view TTA (change vs single-view)
PM on HPMI	AUC	0.987	0.985	−0.002	0.989 (+0.004)
PM on PALM	AUC	0.996	0.993	−0.003	0.994 (+0.001)
MM grading	QWK	0.907	0.907	0.000	0.913 (+0.006)
SE regression	MAE (D)	1.006 (0.873–1.147)	1.259	+0.253	1.264 (+0.005)
Lesion segmentation (interpretation checkpoint)	Mean Dice	0.397 (0.324–0.468)	0.472	+0.075	0.480 (+0.008)

Table S3B. Auxiliary-task comparator results, training-only normalization details, and lesion-specific estimates. Parentheses denote 95% percentile-bootstrap confidence intervals for task-specific baseline predictions. MyoSpectrumNet auxiliary values are legacy aggregate summaries; the between-model gaps are unpaired and descriptive.

Output	Metric	Estimate	Inference/checkpoint	Interpretation
SE baseline	MAE (D)	1.006 (0.873–1.147)	ConvNeXt-T; single-view	Matched-split single-view comparator; baseline 95% CI
SE baseline	RMSE (D)	1.413 (1.204–1.620)	ConvNeXt-T; single-view	Native-diopter output
SE baseline	Pearson r	0.801 (0.746–0.849)	ConvNeXt-T; single-view	Predictions vary with ground truth
SE target normalization	Training mean / population SD (D)	-1.598 / 2.555	992 training records only	Estimated from training records only
SE comparator selection	Selected learning rate / stopping epoch	1×10^{-4} / 18	Training-only internal holdout	Official validation excluded from tuning
SE baseline	Prediction range (D)	-7.32 to 1.67	205-record validation	Not confined to −1 to 0 D
Auxiliary SE	MAE (D)	1.259	MyoSpectrumNet primary; single-view	Legacy aggregate summary; unpaired descriptive comparison
Auxiliary SE	MAE (D)	1.264	MyoSpectrumNet primary; four-view TTA	Within-model sensitivity
Standalone lesion baseline	Overall Dice	0.397 (0.324–0.468)	DeepLabV3-ResNet50; single-view	Matched-split comparator; interval describes baseline uncertainty only
Lesion interpretation	Overall Dice	0.472	Interpretation checkpoint; single-view	Legacy aggregate summary; unpaired descriptive comparison
Lesion interpretation	Overall Dice	0.480	Interpretation checkpoint; four-view TTA	Within-model sensitivity
Standalone lesion baseline	CNV Dice	0.262 (0.047–0.507)	Single-view; n = 7	Lesion-specific estimate
Standalone lesion baseline	Fuchs spot Dice	0.420 (0.323–0.513)	Single-view; n = 13	Lesion-specific estimate
Standalone lesion baseline	Lacquer cracks Dice	0.451 (0.394–0.515)	Single-view; n = 12	Lesion-specific estimate
Lesion interpretation	CNV / Fuchs spot / lacquer cracks Dice	0.250 / 0.544 / 0.545	Interpretation checkpoint; four-view TTA	Secondary TTA context; not primary comparison

Table S4A. Clinical subgroup results. The 'Evaluation records' column is the subgroup evaluation size, not a training-set size. Grades 3-4 contributed 25 records combined; grade-4 row sensitivity was 0.77 for MyoSpectrumNet with four-view TTA versus 1.00 for the task-specific baseline and is considered unstable because of the small subgroup.

Task	Subgroup	Evaluation records	AUC	Accuracy	Secondary metric
PM on PALM	Device 1	353	0.993	0.972	Positive rate 0.487
PM on PALM	Device 2	47	0.994	0.957	Positive rate 0.872
PM on PALM	Center 1	38	0.988	0.921	Positive rate 0.816
PM on PALM	Center 2	284	0.992	0.979	Positive rate 0.408
PM on PALM	Center 3	78	0.995	0.962	Positive rate 0.846
MM grading	Data center 1	215	-	0.819	Grade MAE 0.191
MM grading	Data center 2	33	-	0.879	Grade MAE 0.152
MM grading	Severity 0-1	158	-	0.829	Grade MAE 0.171
MM grading	Severity 2	65	-	0.877	Grade MAE 0.123
MM grading	Severity 3-4	25	-	0.680	Grade MAE 0.440
MM grading	Female	64	-	0.812	Grade MAE 0.188
MM grading	Male	151	-	0.821	Grade MAE 0.192
SE regression	-6 to -3.5 D	57	-	-	MAE 0.802 D
SE regression	-9 to -6 D	42	-	-	MAE 1.896 D
SE regression	<=-9 D	10	-	-	MAE 4.159 D
SE regression	>-3.5 D	96	-	-	MAE 0.960 D
SE regression	Female	104	-	-	MAE 1.248 D
SE regression	Male	101	-	-	MAE 1.280 D

Table S4B. Within-model calibration metrics for raw and temperature-scaled outputs from the same four-view MyoSpectrumNet checkpoint. Lower values indicate better probability reliability. This table does not compare architectures or inference protocols.

Dataset	Output	ECE	Brier	NLL	ECE change after scaling
HPMI	Four-view raw	0.028	0.030	0.124	Reference
HPMI	Four-view temperature-scaled	0.021	0.028	0.100	-0.007
PALM	Four-view raw	0.023	0.024	0.111	Reference
PALM	Four-view temperature-scaled	0.020	0.023	0.090	-0.003

Table S4C. Temperature parameters and empirical high-sensitivity operating points for the same four-view checkpoint.

Dataset	Output	Temperature	Threshold >=0.90 sensitivity	Threshold >=0.95 sensitivity	Comment
HPMI	Four-view raw	-	0.937	0.734	Empirical operating point
HPMI	Four-view temperature-scaled	1.793	0.819	0.650	Temperature fitted on validation predictions
PALM	Four-view raw	-	0.333	0.333	Same observed threshold met both targets
PALM	Four-view temperature-scaled	1.462	0.397	0.397	Same observed threshold met both targets

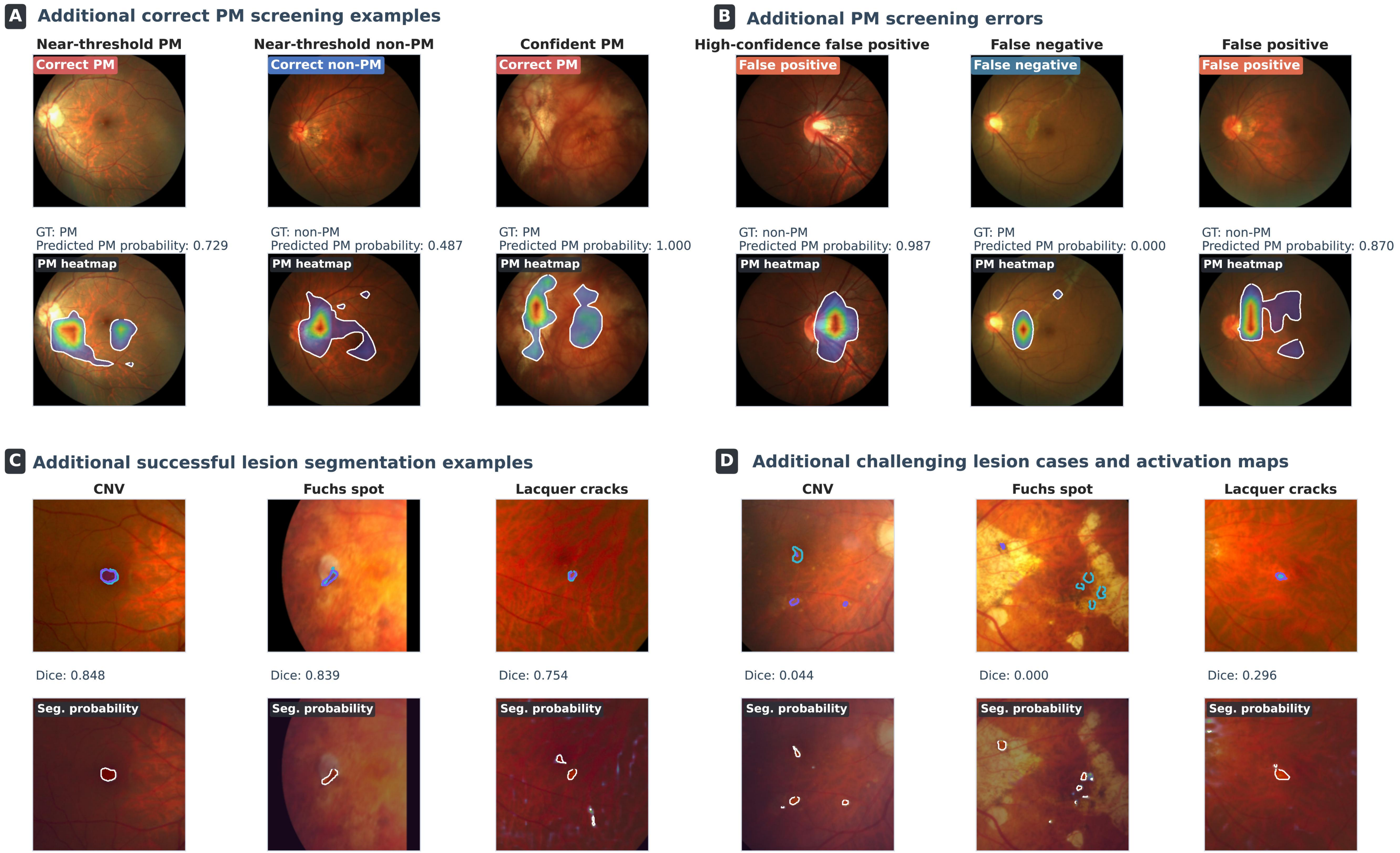

Figure S1. Additional qualitative examples for PM screening and lesion-aware interpretation, including correct, false-positive, false-negative, successful-lesion, and challenging-lesion cases. The examples are illustrative and do not establish comparative superiority.

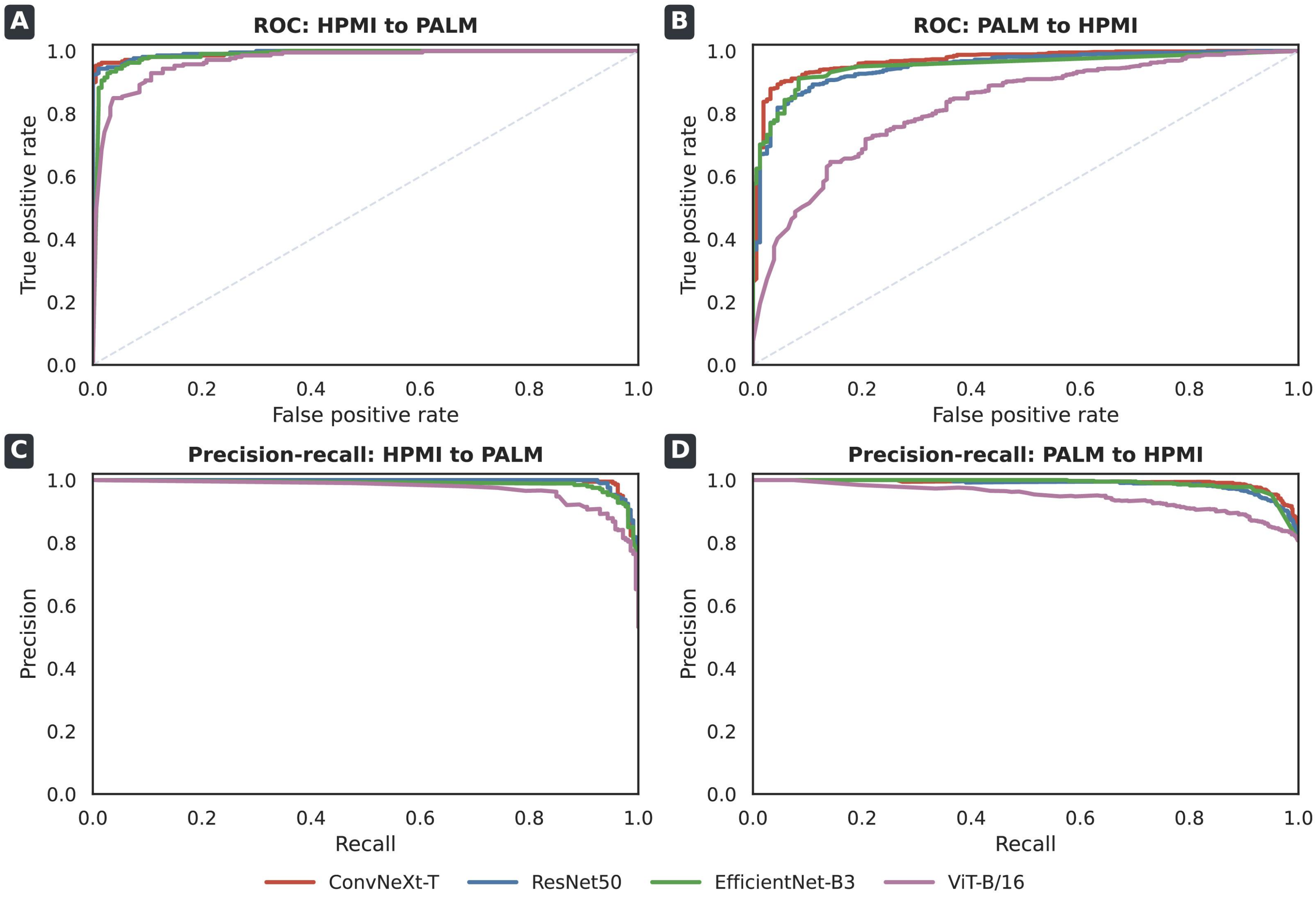

Figure S2. ROC and precision-recall curves for the PM-only HPMI-to-PALM and PALM-to-HPMI source-to-target controls across ConvNeXt-T, ResNet50, EfficientNet-B3, and ViT-B/16. These controls do not use the source-conditioned multi-task checkpoint; they assess PM-screening transportability only and do not externally validate the full four-output framework.

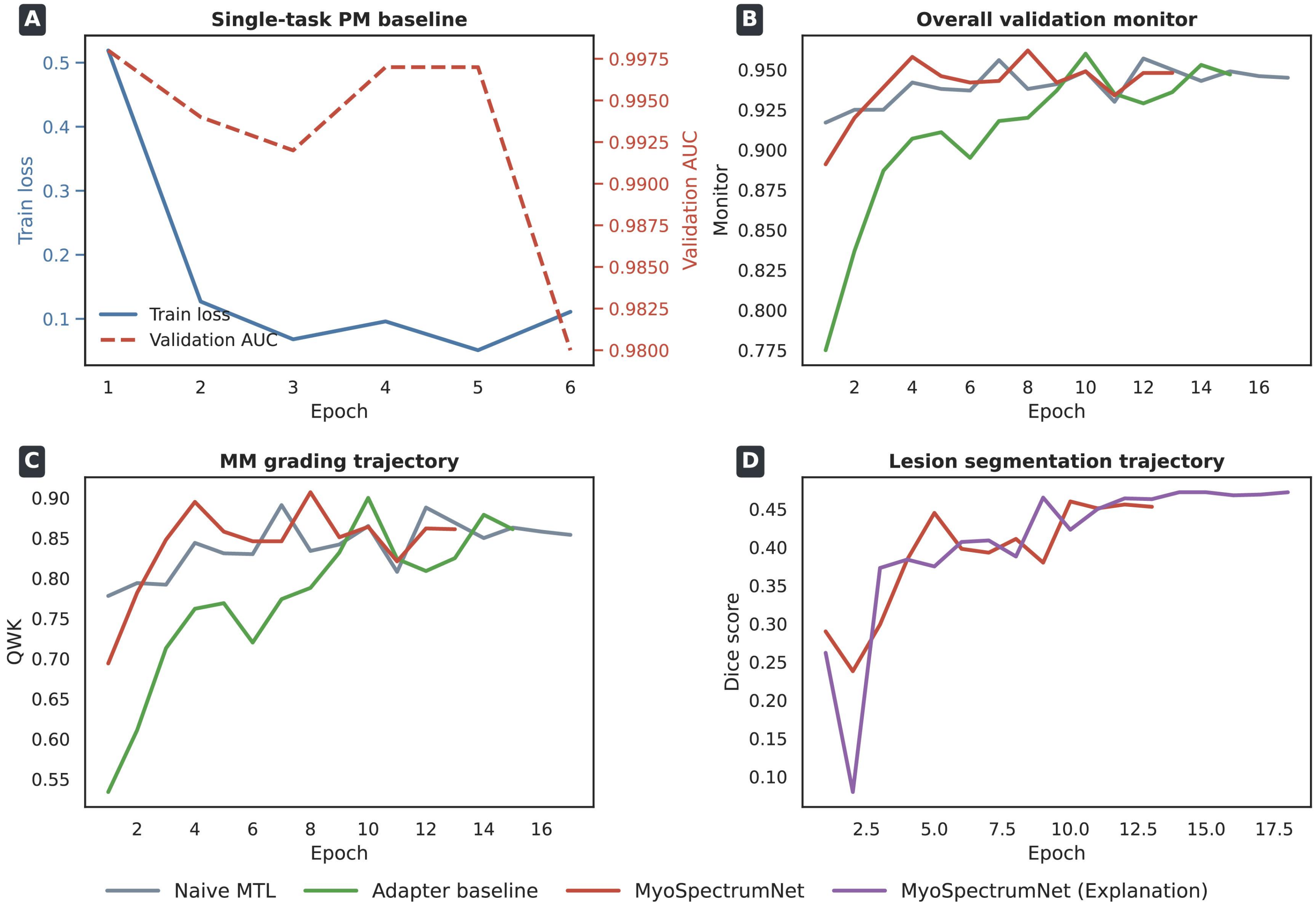

Figure S3. Training dynamics for the PM baseline, overall validation monitor, MM QWK, and lesion Dice trajectories used during model development.

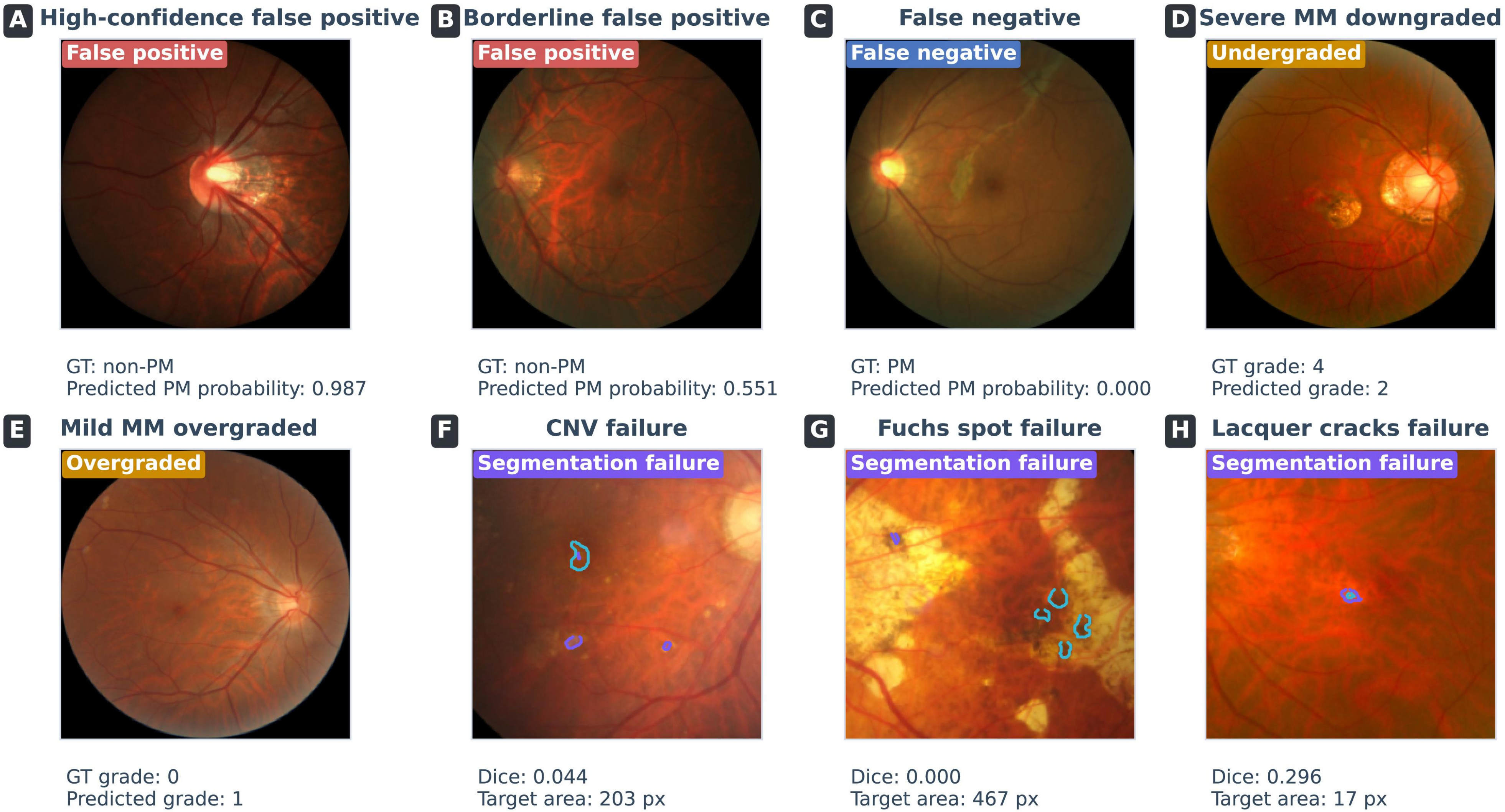

Figure S4. Extended failure analysis for PM screening, MM grading, and lesion-aware interpretation. The examples illustrate boundary ambiguity, advanced structural distortion, and small or low-contrast lesions.

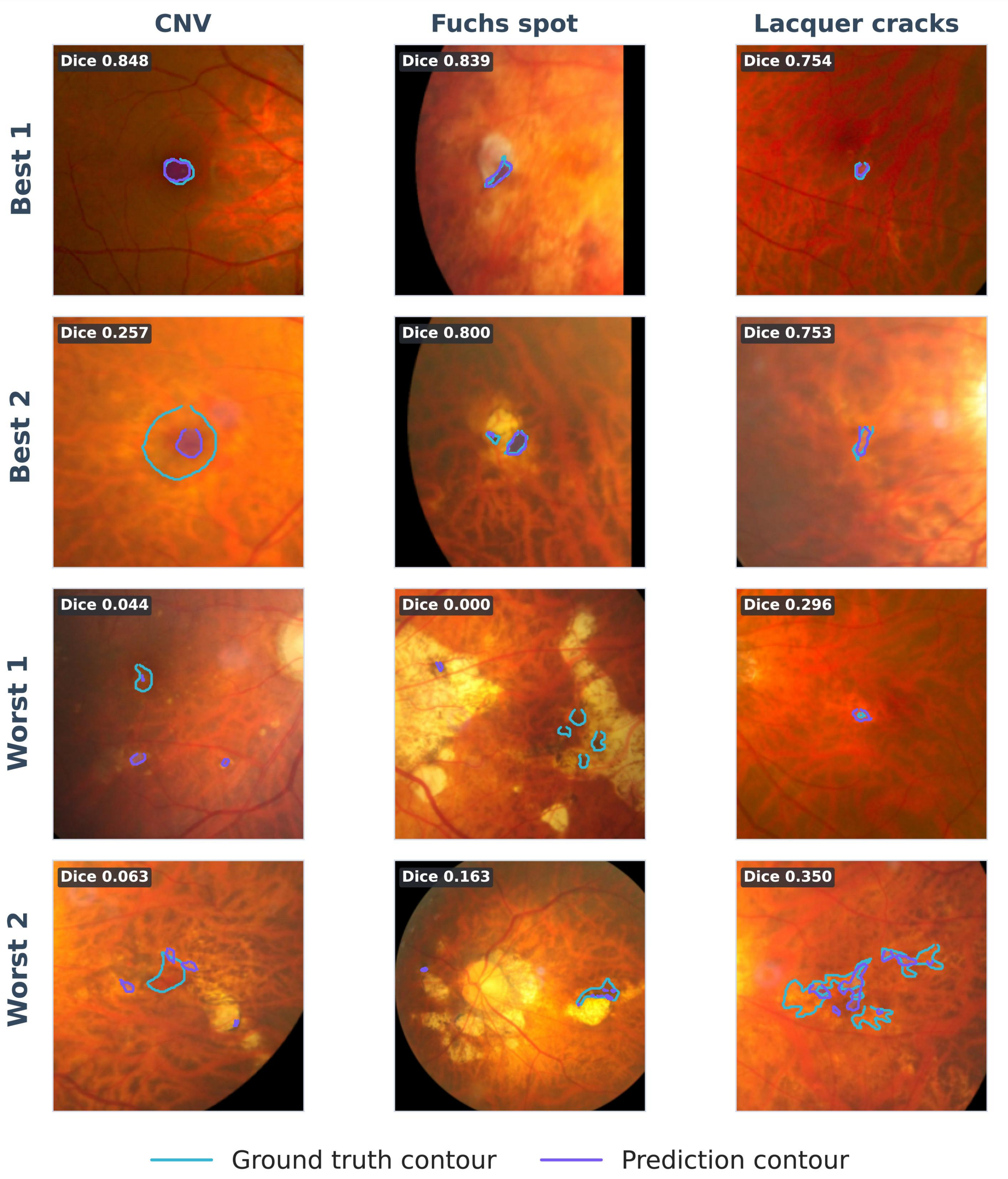

Figure S5. Additional CNV, Fuchs-spot, and lacquer-crack examples across higher- and lower-overlap cases. Cyan contours indicate reference boundaries and purple contours indicate predictions. These qualitative cases do not establish superiority over a standalone segmentation model.
